# The foundation and architecture of precision medicine in neurology and psychiatry

**DOI:** 10.1016/j.tins.2022.12.004

**Published:** 2023-01-13

**Authors:** Harald Hampel, Peng Gao, Jeffrey Cummings, Nicola Toschi, Paul M. Thompson, Yan Hu, Min Cho, Andrea Vergallo

**Affiliations:** 1 Alzheimer’s Disease & Brain Health, Eisai Inc., Nutley, NJ, USA; 2 Chambers-Grundy Center for Transformative Neuroscience, Department of Brain Health, School of Integrated Health Sciences, University of Nevada Las Vegas (UNLV), Las Vegas, NV, USA; 3 Department of Biomedicine and Prevention, University of Rome Tor Vergata, Rome, Italy; 4 Athinoula A. Martinos Center for Biomedical Imaging and Harvard Medical School, Boston, MA, USA; 5 Imaging Genetics Center, Mark & Mary Stevens Institute for Neuroimaging & Informatics, Keck School of Medicine, University of Southern California, Los Angeles, CA, USA

## Abstract

Neurological and psychiatric diseases have high degrees of genetic and pathophysiological heterogeneity, irrespective of clinical manifestations. Traditional medical paradigms have focused on late-stage syndromic aspects of these diseases, with little consideration of the underlying biology. Advances in disease modeling and methodological design have paved the way for the development of precision medicine (PM), an established concept in oncology with growing attention from other medical specialties. We propose a PM architecture for central nervous system diseases built on four converging pillars: multimodal biomarkers, systems medicine, digital health technologies, and data science. We discuss Alzheimer’s disease (AD), an area of significant unmet medical need, as a case-in-point for the proposed framework. AD can be seen as one of the most advanced PM-oriented disease models and as a compelling catalyzer towards PM-oriented neuroscience drug development and advanced healthcare practice.

## Conceptual overview of precision medicine

The term ‘precision medicine’ (PM) has been on the lips and minds of scientists and clinicians alike in recent years. Yet the exact scope and scientific theoretical framework of PM is complex and escapes static boundaries. Despite the landmark announcement of the US Precision Medicine Initiative (PMI) in 2015 [1], how PM should be applied at the individual level, and translated from one disease to another, continues to be debated. The fundamental concept of PM is defined as ‘prevention and treatment strategies that take individual variability into account’ [1]. Despite this seemingly clear-cut definition, the biomedical communities are grappling with the implementation of transformational programs in real-world settings and whether traditionally defined disease entities require redefinition.

The human brain is a highly complex system and is inherently difficult to model due to the dynamic and intricate interactions among its parts. Many of the properties that characterize complex and dynamical systems are relevant in the context of the brain, such as nonlinearity, emergence, spontaneous order, adaptation, and feedback loops. Neurological and psychiatric diseases are often multifactorial, involving different biological systems within a single disease spectrum and resulting from nonlinear interplay of risk genes, dynamic biological determinants, and environmental factors [2–5]. From this complex systems dynamic arise significant individual variabilities in the underlying biology, even when symptomatic and syndromic phenotypes are similar [2–5]. A PM paradigm is pivotal for tackling unmet needs in neurological and psychiatric diseases, which often lack effective treatments and represent a growing burden to healthcare systems and societies worldwide [6,7]. Pharmacological standard-of-care for complex brain disorders is very limited; in the case of brain proteinopathies (including protein misfolding disorders), or pathologically defined ‘primary neurodegenerative diseases’, approved treatments have been mostly drugs with time-limited efficacy and high interindividual variability in response. Moreover, no disease prediction or preventive strategies are available. These issues highlight the need for an evidence-driven revision of the current medical theory and strategies to develop effective biomarker-guided targeted and disease-modifying drugs alongside effective early detection, screening, diagnostic, and therapeutic algorithms.

Reflecting on the modern history of medicine and guided by the framework of the PMI as well as the associated ‘All of Us’ research program (Box 1), here we propose an evidence-based conceptual framework for the transformation to PM in the fields of neuroscience, neurology, and psychiatry. We describe a PM framework as a rational and integrative approach to medical conceptualization, therapy development, and clinical care for multifactorial brain diseases and describe how such a holistic approach can greatly benefit progress in disease characterization and therapeutic development and, ultimately, the individual patient.

## Current models in medicine and their limitations

For a long time, the prevailing model in medicine and drug research and development (R&D) has focused on charting clinically descriptive phenotypic commonalities of large patient populations to identify characteristic signs and symptoms of diseases [4]. This approach falls short of considering the underlying etiology (i.e., genetic and biological dynamics essential to capture the complexity, heterogeneity, and individual progression of neurological and psychiatric diseases). In this context, complexity refers to nonlinear associations, biological crosstalks, molecular mediation pathways [3,8,9]. Another limitation is that past models typically overlooked the long preclinical/prodromal stages of brain diseases, which is arguably the most suitable therapeutic window for recovering and preserving brain homeostasis [3,8,9]. In fact, translational and clinical studies have identified an expanding list of central and peripheral/autonomic nervous systems diseases that are potentially druggable at preclinical and early symptomatic (prodromal) stages. These diseases include, but are not limited to, the traditionally defined cognitive, movement, motoneuron spectrum disorders [e.g., Alzheimer’s disease (AD), dementia with Lewy bodies, Parkinson’s disease (PD), and amyotrophic lateral sclerosis (ALS), as well as idiopathic muscular and peripheral disorders (e.g., Duchenne muscular dystrophy, Charcot-Marie-Tooth disease) and multisystemic disorders (e.g., genetic ataxias and paraparesis)]. Neuropsychiatric and neurodevelopmental conditions such as schizophrenia, autism spectrum disorders (ASDs), anorexia, and suicidality are also examples of major unmet needs, with evidence of the potential for intervention at preclinical stages. An additional level of complexity in neurology and psychiatry clinical research is the methodological constraints posed by the anatomy of the central nervous system (CNS), which precludes regular tissue biopsies. Because of these limitations, hypotheses regarding etiology, pathogenesis, and pathophysiological mechanism(s) of CNS diseases are often based on *a priori* assumptions and precipitous translation of preclinical models to human research. For instance, translational models of the neurodegenerative disease spectrum indicate a prominent role of inflammatory and immune responses in the pathophysiological process, and preclinical data support a potential role for anti-inflammatory treatments in AD and ALS, among others [10]. Of note, clinical trials with non-steroidal anti-inflammatory drugs have failed to prove efficacy so far [11] (detailed examination of the potential factors accounting for the lack of success are beyond the scope of the discussion here). Recently, clinical research paradigms and blueprints have introduced systematic assessment of biomarkers, which broadened the understanding of the molecular mechanisms behind neuroinflammation. Such implementation has facilitated the development of promising compounds [9,12].

Clinical evidence suggests that neurological and psychiatric diseases often transcend the strict dichotomous distinction between health and disease. Rather, health and disease exist in an evolving dynamic continuum, especially for conditions that do not follow a linear course (of continuous progression) and that involve a chronic natural history. During the preclinical stages of these diseases, genetic, environmental, and stochastic factors trigger and drive aberrant biological pathways that unfold at different rates across genetic-, epigenetic-, molecular-, cellular-, tissue-, and macro-scale networks, while relevant physiological functions exhibit only subtle changes due to compensation mechanisms at different biological system levels [3,5,13,14]. For instance, studies in AD suggest that the early preclinical stages have homeostatic and cellular adaption mechanisms that afford resilience to the incipient pathophysiological changes, a compensation mechanism that may be lost as the disease progresses [15].

Along the continuum of health and disease, there is a prodromal phase when pathophysiological changes become detectable and syndromic phenotypes start to manifest. At this point, homeostasis, with the underlying core biological feedback loops and networks and systems have been overwhelmed and begin to break down at varying points along the spatial and temporal continuum with decompensation and subsequent system failure in an individual manner [16,17]. This process culminates in an ultimate and potentially irreversible multiscale system failure stage (i.e., the clinically overt late phase of the disease). During this stage, therapeutic intervention is increasingly unlikely to substantially modulate biological pathways and pathophysiology and has little chance to produce significant and meaningful benefits in patients [9,18–20].

## PM: the rise of a paradigm shift and the pioneering model of oncology

PM is an emerging translational science paradigm related to the evolutionarily developed, complex multidimensional health–disease homeostasis and continuum, which aims to optimize the effectiveness of disease prevention. It deploys time-sensitive detection/diagnosis and treatment strategies tailored to the individual’s specific clinical–genetic–biological characteristics, psycho-social environment, and lifestyle risk factors [1]. Such a holistic healthcare approach is actionable only through deep understanding of the clinical–biological trajectories of disease and the identification of at-risk populations. Following this crucial step of clinical research, the development of stage-dependent and pathway-based therapies that target critical causal factors and upstream molecular and cellular alterations can be attained. Eventually, PM-oriented strategies are hoped to lead to effective integration of nonpharmacological (i.e., lifestyle-related) interventions and individualized pharmacological treatments, for primary and secondary prevention and treatment of asymptomatic preclinical and prodromal disease stages.

**Figure 1.**
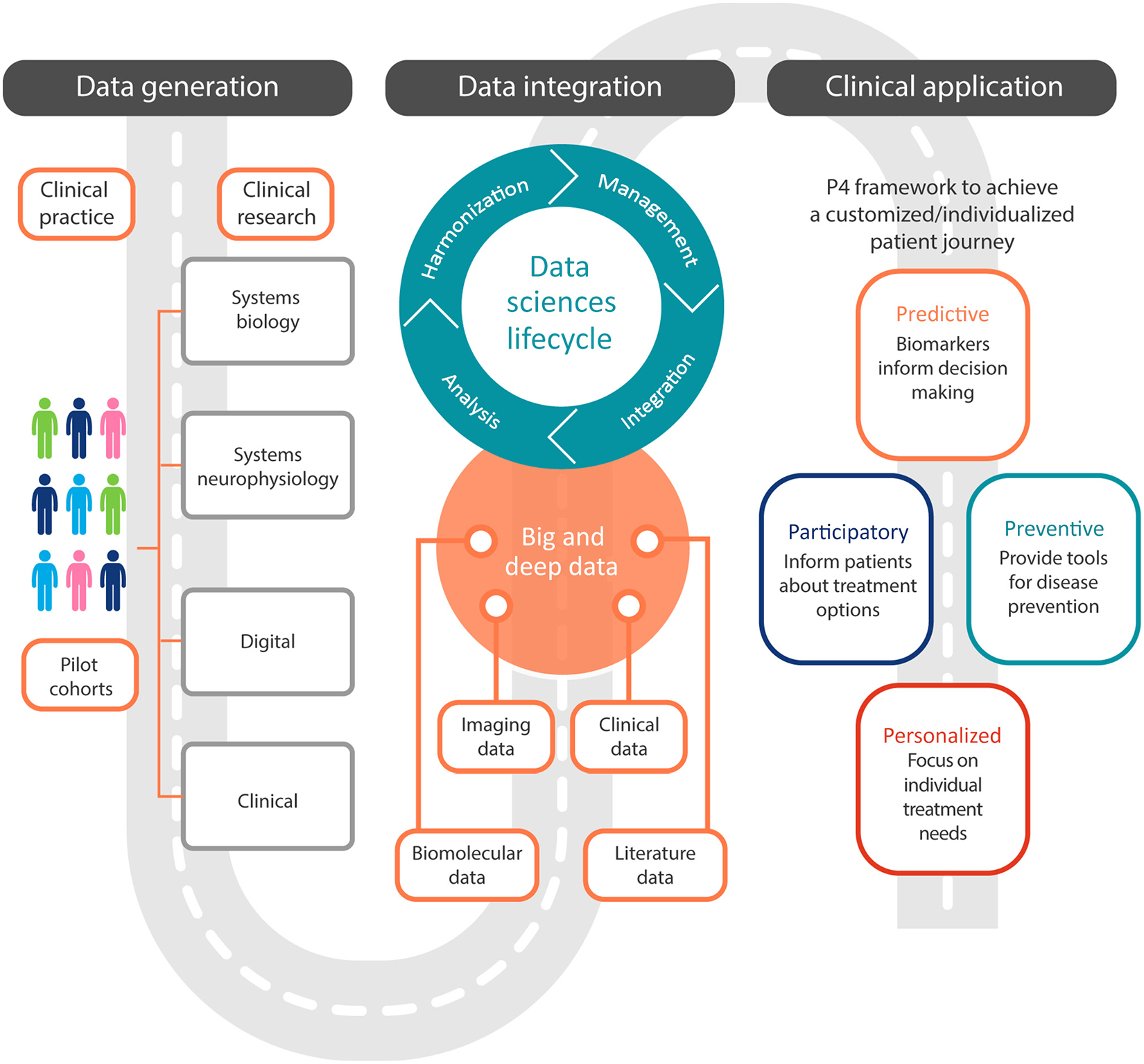
The road to precision medicine (PM) in neurology and psychiatry: towards predictive, participatory, preventive, and personalized (P4) medicine and optimized patient journey. The P4 paradigm envisions a healthcare landscape based on the elements of predictive, participatory, preventive, and personalized medicine [21,90]. The framework outlined in the current article aims to present a path for deploying the P4 paradigm in the fields of neurology and psychiatry. As summarized in the figure, the proposal is grounded on four converging pillars: systems medicine, digital technologies, biomarkers, and big data. Information is gathered from large populations to provide personalized medicine for individuals with neurological and psychiatric diseases. Digital and clinical data generated through systems medicine are gathered and integrated to create big and deep data. A structured data science approach is used to integrate complex data and provide meaningful outputs. This is the necessary substrate to support the P4 framework. This framework integrates the four Ps with the ultimate goal of prolonging health span through early interventions.

In its full deployment, PM in clinical practice will embrace the ‘P4’ medicine paradigm: (i) stratification of individuals based on the risk of developing the disease (predictive); (ii) large-scale screening and early detection for timely therapeutic interventions (preventive); (iii) tailoring treatment(s) to the patient’s social–clinical–biological characteristics (personalized); and (iv) optimizing ‘actionable’ plans to benefit all patients through patient-centered individualized data collection and utilization such as self-monitoring and self-assessments (participatory) [21,22]. PM would ultimately enable an individualized healthcare workflow and patient journey, skewing the curve of resource investment and success towards prolonging health span over disease management (Figure 1).

The field of oncology has pioneered the development and implementation of PM-oriented and patient-centered approach in research and clinical care [23]. In the past few years, the US FDA authorized molecular pathway-targeting drugs that can be used in a tissue- and tumor-agnostic fashion. This disruptive innovation has been achieved through exploratory and systematic biomarker profiling studies, identifying the critical (epi)genetic and biological factors rather than focusing solely on traditional assessments such as histology and organ site (Box 2) [24,25]. Capitalizing on key concepts and operating models successfully employed in oncology, one can envision implementation of the PM framework in neuroscience indications, neurology, and psychiatry as well.

## The pillars of PM in neurology and psychiatry

Building on the successful oncology model, we propose an evidence-based conceptual architecture of PM in neurology and psychiatry that is built on four pillars: (i) biomarkers, (ii) systems medicine, (iii) digital technologies, and (iv) data science (Figure 1). This conceptual framework could support the process of redefining diseases according to clinical–biological constructs embedded in a continuum and, crucially, allow the identification of the preclinical stage, a critical time window when restoring brain network homeostasis and prolonging the brain health span are most feasible.

## Biomarkers as a multidimensional description of pathophysiological alterations for different contexts-of-use: genetics and single-/multi-omics profiling

The identification of genetic variants contributing to Mendelian CNS diseases has already transformed clinical care towards genetically informed diagnostic and therapeutic decision-making. One example is deficiency of the *SMN1* gene that results in spinal muscular atrophy [26]. In various neurological and psychiatric conditions, highly penetrant causal variants and risk genes have been identified. This includes monogenic forms of AD or frontotemporal dementia [27], PD and other movement disorders [28,29], ALS and other motoneuron disorders, schizophrenia [30], and ASD [31]. Insights obtained from genetic studies have provided the crucial entry point and helped identify key biological/pathophysiological processes underlying subtypes of these complex diseases [32].

However, familial forms of neurological and psychiatric diseases, caused by hereditary genes, represent only a small fraction of the total disease cases. For the vast majority of patients, genetic risk reflects the cumulative impact of common genetic variants that individually exert a small effect on disease susceptibility [27,28,33]. Large-scale population genomic analyses, such as genome-wide association studies (GWAS), have identified common genetic variants associated with several clinical phenotypes in neurology and psychiatry [33–37]. Importantly, genetic overlap of common brain diseases is increasingly recognized. These observations indicate the presence of highly conserved molecular pathways linked to specific clinical manifestations and pathophysiological commonalities and corroborate findings from experimental models showing that chronic, clinically heterogeneous diseases of the CNS unfold across multiple biological levels and systems [38]. Experimental and clinical evidence indicates that the genetic architecture of neurological and psychiatric diseases can involve pathways that extend beyond the CNS. In this regard, crosstalks between the periphery and CNS have been reported in the context of the immune and inflammatory responses, lipid and glucose metabolism, and functional regulation of the glymphatic and blood–brain barrier systems [34,35,39].

The development of polygenic risk scores (PRS), a combination of genetic variants weighted by their effect sizes, has provided opportunities for translating genomic findings to clinical care [40,41]. Recent studies of AD, schizoaffective disorder, and ASD have shown that PRS can identify individuals with increased susceptibility or risk levels [36,42–45]. Moreover, PRS studies can support investigation of covariance between clusters of genetic factors and clinical (endo)phenotypes [46,47]. Although at present PRS is still used only for research purposes, it is conceivable that in future clinical practice, PRS may inform screening, therapeutic decision-making, and the deployment of preventive strategies [48].

Omic integrative methods that bridge genomics, phenotypes, and function offer an unprecedented opportunity to obtain insights into disease mechanisms and to accelerate the discovery of molecular biomarkers [49,50]. Epigenomics, the systematic investigation of nonmutational gene expression patterns within the genome, provides a means to systematically explore the effects of gene–exposome interaction [51]. Epigenome-wide association studies point to various gene-regulatory mechanisms and environmentally induced post-translational modifications that account for mechanistic alterations and biological heterogeneity in sporadic diseases [52]. Transcriptomics explores the broad set of RNA transcripts; clinically relevant gene expression signatures of different neurological and psychiatric diseases are being mapped out [53]. Transcriptome-wide association studies have the potential to supply meaningful insights into the spatial and temporal coordinates of causal and secondary mechanisms linked to newly identified genetic and biological risk factors [10,54,55]. Proteomics has been widely used to identify the ultimate pathophysiological mechanisms as well as to develop, validate, and qualify bodily fluid biomarkers in AD, PD, and schizophrenia [56–58]. Albeit still more preliminary than other omic layers, metabolomics and lipidomics hold the potential to provide highly individualized information about bioenergetic, metabolic, and lipid homeostasis processes, relevant to critical pathophysiological pathways that occur in neurological and psychiatric disorders [59–61].

### Bodily fluid matrixes for biomarker assessment

Various bodily fluids, including cerebrospinal fluid (CSF), blood (plasma, serum), and more recently saliva and urine, have been used as a source to develop biomarkers for different contexts-of-use in several neurological and psychiatric conditions [62–64]. Fluid biomarkers for brain diseases are particularly attractive as they circumvent the physical constraints imposed by the brain’s anatomy for research and healthcare practice (encapsulated in the concept of liquid biopsy, Box 3) [65]. Fluid biomarker analysis also enables simultaneous investigation of multiple biological alterations, which is pivotal for complex diseases with multifaceted pathophysiology and dynamic temporal profiles.

Traditional biomarker discovery has relied on translational research and animal models. Omics science not only facilitates and boosts the accumulation of knowledge about genetics, risk factors, and molecular pathways underlying the biology of neurological and psychiatric diseases in humans [53], but also accelerates the identification of candidate biomarkers. Summarizing advances in this evolving area would be beyond the scope of the current article and we refer readers to recent review articles that have addressed the topic, particularly in the context of neurodegenerative disorders [66–68].

### Neuroimaging biomarkers

Neuroimaging, including molecular and structural/functional imaging, allows noninvasive visualization of the CNS and supplies both qualitative and quantitative data. Molecular imaging methods, such as positron emission tomography (PET) and single-photon emission computed tomography (SPECT), use radio-ligands that bind to distinct molecular targets implicated in disease-relevant biological pathways. Molecular imaging can directly detect disease-associated molecular and cellular process(es), such as protein misfolding and accumulation (e.g., measured by amyloid and tau PET) [69,70], changes in neuronal metabolism (e.g., measured by fluorodeoxyglucose PET), microglial activation (e.g., detected by translocator protein [TSPO] imaging) [71], and neurochemical dysfunction (e.g., measured using cholinergic, glutamatergic, or dopaminergic system radiotracers) [72,73].

The tight association between the uptake of certain radiotracers and corresponding neuropathological findings has generated core/supportive diagnostic biomarkers for different neurodegenerative diseases [73,74]. Moreover, recent quantitative approaches, leveraging automatic analysis pipelines, allow the *in vivo* tracking of neurobiological pathways, resembling the traditional neuropathological staging and potentially supporting stage-driven therapeutic approaches [69].

Magnetic resonance imaging (MRI) provides a window into the structural and functional organization of the brain. Structural MRI captures cortical and subcortical grey matter volumes, shapes, and surfaces, as well as white matter connections and microstructural properties. Functional MRI reveals activation patterns, including functional integration or segregation among brain areas/networks, both at rest and during cognitive tasks. The resulting maps of brain activity patterns, when combined with structural information, may serve as a fingerprint for each patient [75,76].

Some of the frontiers in human neuroimaging include the development of spatio-temporal maps of short- and long-range connections (connectomes), integration of structural and functional data (structural–functional connectivity coupling), and characterization of the modular organization of the brain. These advances hold the promise to reveal biomarkers in the form of subtle changes in the hierarchical organization of the brain that may underlie altered cognition and behavior [5,77]. Work from recent years has identified structural and functional brain endophenotypes of typical cognition, behavior, and movement, as well as related alterations in neurological and psychiatric diseases [78–80]. For example, the resting state functional connectome of the brain has shown promise in differentiating individuals with specific neurodevelopmental conditions (e.g., ASD) from typically developing controls [80] and predicting an individual’s response to treatment in various mental disorders (e.g., anxiety [79] or depression [78]).

Changes in regional and whole-brain functional architecture on the millisecond time scale may reflect physiology- or disease-related alterations in the brain [81–84]. In view of the low temporal resolution of MRI, this methodology can be complemented by electroencephalography (EEG) and magnetoencephalography (MEG), which offer noninvasive albeit indirect assessment of neuronal activity at high temporal resolution. Recent efforts have merged genomic and EEG technologies to discover genomic variants that affect brain synchrony, offering new mechanistic insights into genetic variants associated with alcohol use disorders and epilepsy (see Outstanding questions) [85].

## Systems medicine

Critical biological factors whose perturbation may lead to systems failure can be uncovered by computational analysis of large, multidimensional datasets under the systems-network theory [86–88]. Basic and high-level properties of key nodes and modules can be mapped, in static and dynamic conditions, to decipher causative genetic–biological dynamics before they lead to an overt phenotype [86–88]. This provides a mechanistic entry into the complex genetic and pathophysiologic landscape that underlies disease signs and symptoms. For neurological and psychiatric diseases, systems biology and systems (neuro)physiology can provide comprehensive models of structural and functional organization of the brain in health and disease [85,89].

### Systems biology: an overview

We use the term systems biology to refer to approaches aimed at description and quantification of the relationship between molecular biological levels of a given system and for methods intended to generate explainable readouts of causative dynamics, intermediate endophenotypes, or clinical features. According to the concepts in systems biology, physiological functions and pathophysiological changes may be mapped along highly connected networks of genes/proteins/metabolites/lipids with critical connection and intersection [49,53,85,90,91].

**Figure 2.**
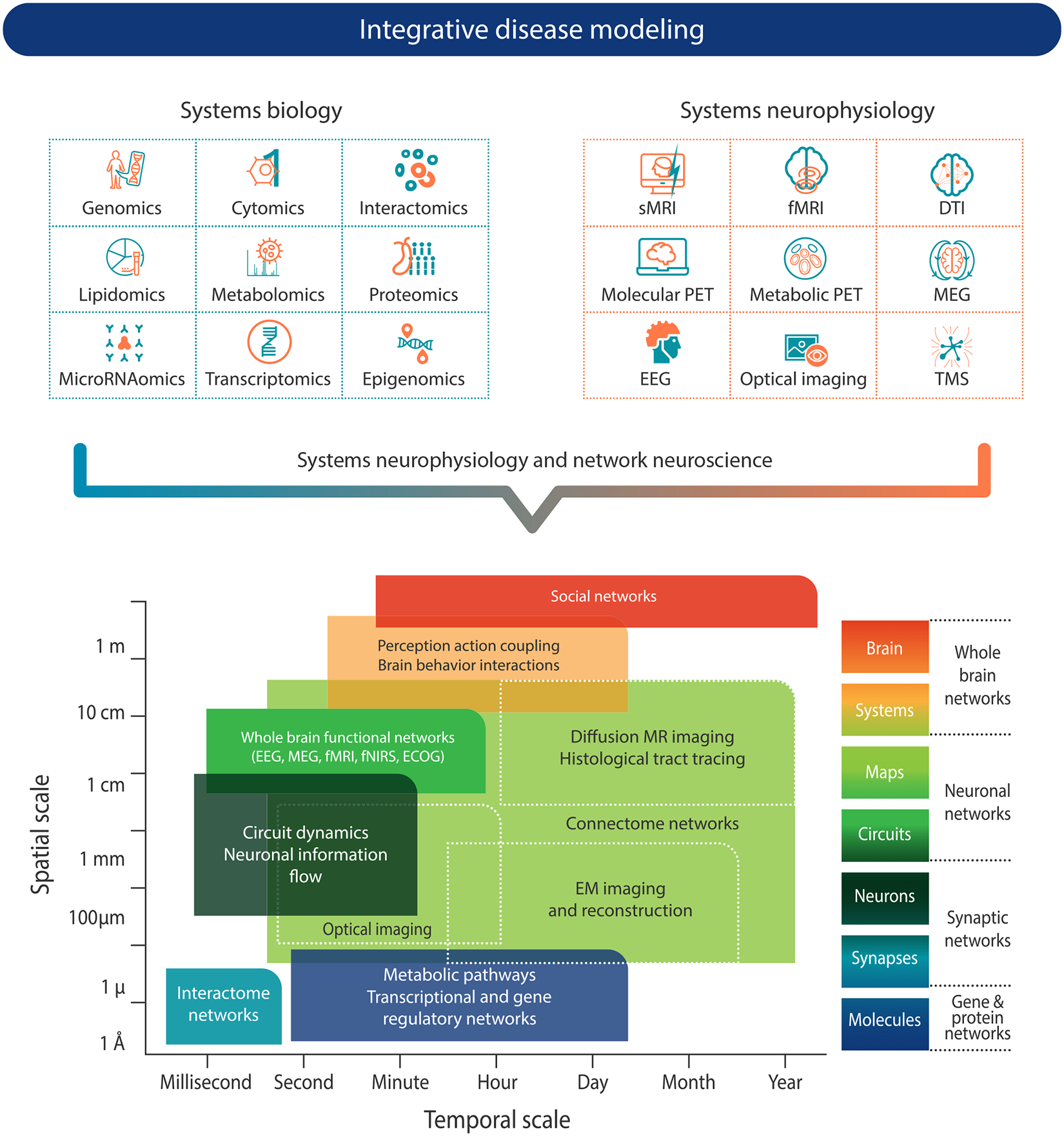
Systems biology and systems neurophysiology data provide information across different spatial and temporal scales. Multiple types of data can be obtained from systems biology, including quantification of neurobiological systems at the molecular biology level, and systems neurophysiology, which encompasses multimodal integrative imaging or recording techniques to capture data at different spatial and temporal scales. These data can be integrated for the purpose of systems modeling across spatial and temporal ranging from the atomic and molecular scale to whole brains, and from millisecond-range phenomena to processes progressing over years. Abbreviations: DTI, diffusion tensor imaging; ECOG, electrocorticogram; EEG, electroencephalography; EM, electron microscopy; fMRI, functional magnetic resonance imaging; fNIRS, functional near infrared spectroscopy; MEG, magnetoencephalography; PET, positron emission tomography; sMRI, structural magnetic resonance imaging; TMS, transcranial magnetic stimulation.

Progress in systems biology has been fueled by the recent advances in high-throughput omics science, data mining and modeling approaches, and by the development of accessible classification tools for functional annotation [92,93]. In neurological and psychiatric diseases, omics and multi-omics profiling and systems biology have been widely applied to brain tissues and biofluid samples to gain understanding of disease pathophysiology and dynamics and to identify potential biomarkers (Figure 2). The various omics layers have been described in an earlier section (‘Biomarkers as a multidimensional description of pathophysiological alterations for different contexts-of-use: genetics and single-/multi-omics profiling’). As a next step, multi-omics integration could allow different biological organizational levels to be explored simultaneously, resulting in a holistic understanding of genetic-driven or stochastic changes in the CNS [94–96]. For example, genomic, tissue-level and single-cell transcriptomics and epigenetic data have been integrated to identify gene regulatory networks in the brain and predict endo- and syndromic phenotypes of psychiatric disorders [97]. Exploratory systems biology approaches have also been used to map gene-to-phenotype and protein-to-phenotype connections, identifying shared etiologies among different diseases [38].

### Systems neurophysiology: multimodal integrative monitoring of neural activity at different spatial and temporal scales

Systems neurophysiology, as defined here, aims to integrate structural and functional brain activity features across different spatio-temporal scales to generate a functional atlas of neural activities throughout development and aging and in health and disease (Figure 2) [98]. Examples include the reconstruction of the hierarchical organization of the brain in young and aged individuals with normal cognitive/motor functions [99,100] and in several neurological or psychiatric diseases, including AD [101–104], PD and parkinsonism [105], and schizophrenia [106,107].

The combination of molecular imaging (PET/SPECT) and structural and functional imaging (MRI, fMRI, EEG, MEG) has been refined to show spatial-temporal association of protein aggregate accumulation or regional neurochemical/metabolic alterations overlaid with cortical/white matter damage, network functional activity changes and clinical features [99–102,106,108–113]. This multimodal and multidomain combination allow all of these factors to be investigated jointly and integrated in a system-wide manner, providing insights into their interplay and modulation and how such interplay may become aberrant in neurological and psychiatric diseases [99–102,106,108–113].

**Figure 3.**
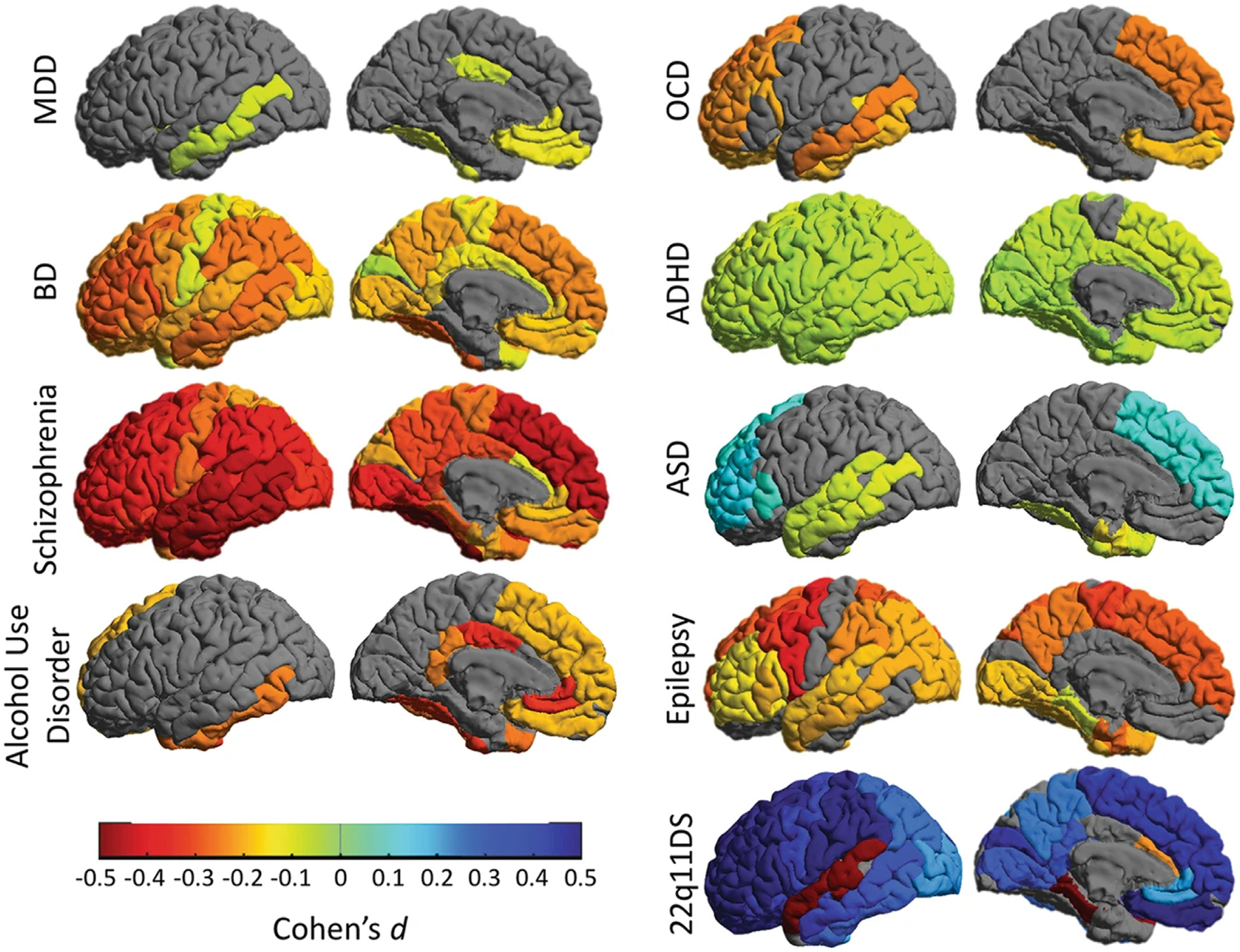
Large neuroimaging studies of several major neurological, psychiatric, and developmental conditions reveal both overlap and characteristic differences in the profiles of brain alterations. Findings depicted in the figure are by the ENIGMA Consortium [19]. Cortical grey matter thinning is prevalent in a range of conditions examined in the study, except for autism spectrum disorder and 22q11 deletion syndrome, where excess brain tissue is found. Recent work has related some of these patterns to cell-specific gene expression patterns and to neuroreceptor distributions [114], implicating specific cell types and molecular pathways in psychiatric conditions. Reproduced from [19]. Abbreviations: 22q11DS, 22q deletion syndrome; ADHD, attention deficit hyperactivity disorder; ASD, autism spectrum disorder; BD, bipolar disorder; MDD, major depressive disorder; OCD, obsessive-compulsive disorder.

New links from multimodal brain imaging to cellular and molecular data have recently been established by the ENIGMA Consortium. Established in 2009, the consortium conducted the most extensive neuroimaging investigations of several major neurological and psychiatric conditions, from PD, epilepsy, and ataxia to schizophrenia, bipolar disorder, depression, substance use disorders, and post-traumatic stress disorder (Figure 3) [19]. In an approach termed ‘virtual histology’, the characteristic patterns of imaging abnormalities across diverse brain disorders appear to relate to several molecular and cellular features: (i) transcriptomic data and gene expression patterns mapped in the Allen Brain Atlas, and (ii) neurotransmitter distributions mapped in a normative atlas of 18 receptors and transporters across nine different neurotransmitter systems [114]. The ENIGMA Toolbox, developed to compare brain disorders with each other and with histologic and molecular data, has facilitated the discovery of specific cell types and systems that may be implicated in major psychiatric conditions, offering new mechanistic leads for research in psychiatry [115].

Brain activity characterized solely in the form of anatomically segregated responses is insufficient to explain the complexity of neurodevelopment, cognition, behavior, aging, and related diseases [116]. A higher order statistical analysis and network-level concept is needed to uncover potential sources of neural and glial dysfunction. In the past two decades, graph theoretical measures applied to neuroimaging have revealed abnormalities of network configurations in clinically defined pathological conditions [89]. One such effort integrated neuroimaging and connectome analysis to identify network associations with atrophy patterns in 1021 adults with epilepsy compared with 1564 healthy controls from 19 international sites; this work identified disease epicenters and hubs, intrinsic features of brain networks that helped explain the patterns of atrophy seen across multiple epilepsy syndromes [117].

## Digital health devices and technologies

Neurological and psychiatric diseases often manifest in several physiological systems and functional domains such as changes in complex behaviors, social interactions, and sleep patterns. Digitally enabled data collection may capture the rich and diverse repertoire of disease-related phenotypes that cannot be readily assessed during clinical visits alone [118]. Digital health technologies hold the unique advantage of being portable and intrinsically quantitative, allowing data collection to be convenient, unobtrusive, and longitudinal. Digital health data can span a multitude of biometrics related to central and peripheral autonomic system functions (e.g., heart rate, body temperature, cardiac rhythms, skin conductance, blood oxygenation) and cover clinically relevant parameters (e.g., motion, gait, pace, sleep, speech and voice patterns) [119,120]. Digital technologies can detect subtle changes during early stages of disease, offering solutions for screening and early diagnosis. They also open new possibilities for longitudinal data collection and have the potential to provide useful information on prognosis and disease progression [118].

There has been a steep increase in pilot studies, multicenter clinical trials, and large-scale observational datasets exploring the performance of various digital health devices that could provide surrogate measures for clinical outcomes [119–121]. The field of movement disorders as traditionally defined has dramatically benefited from digital biomarker development programs; wristwatch-type wearables and smartphones with built-in accelerometer and gyroscope can capture aspects of tremor, bradykinesia, dystonic movements, and impairments in gait and balance [119,122–124]. More broadly, actigraphy, other wearable analytics, and smart technologies are under development to support early detection and management of different behavioral and psychological symptoms, such as psychosis, changes in mood, and circadian rhythm disruption, in several neurological and psychiatric conditions [125–128]. In addition, digital technologies are being explored as therapeutics [129].

## Data science

Generating ‘big data’ is an inevitable outcome of current technology trends, as technologies are evolving to capture increasingly comprehensive datasets of physiological and behavioral measures from individuals [omics data, brain structural and functional data, continuous health data from wearables, electronic medical records (EHRs), etc.]. Massive in quantity and complex and heterogeneous in nature, big data can be challenging to analyze using traditional statistical approaches. Computational models based on artificial intelligence (AI) approaches can generate clinically meaningful readouts using sparse and noisy multidimensional data from different sources [130]. The widespread use of machine learning (ML), especially in the development of deep learning (DL) algorithms, has revolutionized the application of AI in clinical research and drug R&D [131]. DL methods have been developed to detect AD based on learning patterns in MRI scans from over 200 sites worldwide [132]. Other approaches have synthesized novel image contrasts [133], boosted scan resolution and speed [134], and even learned to infer neuropathology from *in vivo* scans not previously thought to be sensitive to such molecular features [132].

Owing to its predictive abilities, AI is expected to facilitate the time-dependent analysis and serial/longitudinal tracking of patients’ clinical and medical data-rich profiles. AI algorithms may support medical data aggregation and filtering, as well as clinical decision-making based on manually curated data (i.e., supervised learning). In addition, AI may help identify clinically relevant subgroups of individuals (e.g., genetic–biological clusters) from large, heterogeneous populations who at the surface level share clinical phenotypes or disease labels [131–133]. This can be accomplished by autonomously searching for association within the high dimensional data space (i.e., unsupervised learning) [132,133]. The resulting clusters or latent dimensions of variation can in turn reveal the latent, long-postulated biological heterogeneity underlying the symptoms that may influence treatment response and clinical–biological trajectories [131,135]. This approach could be applied to AD, other neurodegenerative diseases, and a spectrum of affective disorders [78,136–139]. Unsupervised AI approaches can also finely dissect preclinical stages of diseases to uncover hidden biological signatures [78,140–142]. In clinical trials, unsupervised algorithms trained on clinical or biomarker data have already been shown to predict treatment response in depression and AD [142,143]. The near universal adoption of EHR across healthcare systems has enabled the collection and storage of large, population-wide real-world clinical data in a digital format that can be systematically analyzed. Analysis of EHR databases with AI has augmented diagnosis, prognosis, and prediction of disease onset or progression to better inform clinical decision-making [144]. Such models, once trained, could offer a relatively low-cost and scalable alternative to traditional population screening to identify high-risk populations who should be further evaluated with more specific testing [145,146].

In summary, there is a global multidisciplinary effort to implement applied AI techniques across translational/clinical/pharmacological research areas and medical practice. Besides facilitating PM-oriented drug R&D, AI-assisted medicine promises to significantly lower time and resource investment for healthcare infrastructures by streamlining screening, diagnostic, and therapeutic pathways. Although significant progress has been made recently, various challenges, including the need for explainability and trustworthiness [147], hinder the AI-scaled transformation of medicine and neurology. Human-readable physiological insights may facilitate adoption by clinical practitioners. Lastly, ethical aspects of the use of AI in biomedical research and medicine require careful consideration and are being tackled with dedicated approaches [148].

## Potential limitations and challenges for big data approaches in PM

While AI approaches can deliver high performance, a key limitation is that little or no insight may be gleaned into the inner workings of these models (the ‘black box’ issue). This often limits our understanding of how data have influenced model output [149]. A related point is that AI methods may reveal systems complexity; while recognizing this complexity is an essential step towards understanding the disease state(s) and the compensation to incipient dynamics that prevent systems failure, current AI approaches are limited in elucidating how or why complexity arises, thus making interpretation and clinical decision difficult [149]. Another challenge is the need for an input dataset with consistent curation and harmonization; diverse and rich datasets need to be available to reflect multiple dimensions of health and disease as highlighted for neuroimaging big data analytics [150].

Many of the challenges in applying AI methods arise at the level of systems biology. The challenges become more acute when transitioning from domains such as gene–gene association analyses, protein–protein coexpression networks, or metabolomic pathway analyses, to layering multi-omics analysis. Multimodal, integrative, and systems-scale paradigms hold the potential to map clinical–biological trajectories of brain endophenotypes in cognitively healthy individuals at risk; for instance, in the context of AD, carriers of the apolipoprotein E (APOE) e4 allele, individuals with incipient Aβ/tau accumulation, or people reporting subjective memory complaints [5,53,62,151]. However, the need for data standardization and curation in large and automated analyses is particularly relevant when overlaying different big data sets. This complexity likely plays a part in the relatively limited implementation of large-scale, multimodal data collection and monitoring in AD. While in presurgical assessments of epilepsy and neuro-oncology, combined functional imaging (fMRI-EEG coregistration) is routinely used, the use of multimodal neuroimaging in late-onset AD, especially fMRI and molecular brain mapping of amyloid and tau using PET, is still far from widespread clinical implementation, partly due to the cost and complexity of these techniques.

Standardization of AI algorithms in drug R&D and healthcare requires more in-depth analytical and clinical validation. Translating complex systems biology and neurophysiology outputs into reliable, reproducible, and operable data for drug R&D and healthcare decision-making requires user confidence and significant investment to apply the information to patient and physician needs. Another potential issue concerns the capability of AI-based processes in weighting non-clinical factors of individual patients, such as health–economic aspects that play an important role in the P4 framework and healthcare in general. Algorithms that are agnostic to the patient’s socioeconomic status, access to healthcare, and social determinants of health, may generate infeasible healthcare journeys, thus delaying the diagnosis and management of disease.

## AD: a blueprint towards PM in neurodegenerative diseases

**Figure 4.**
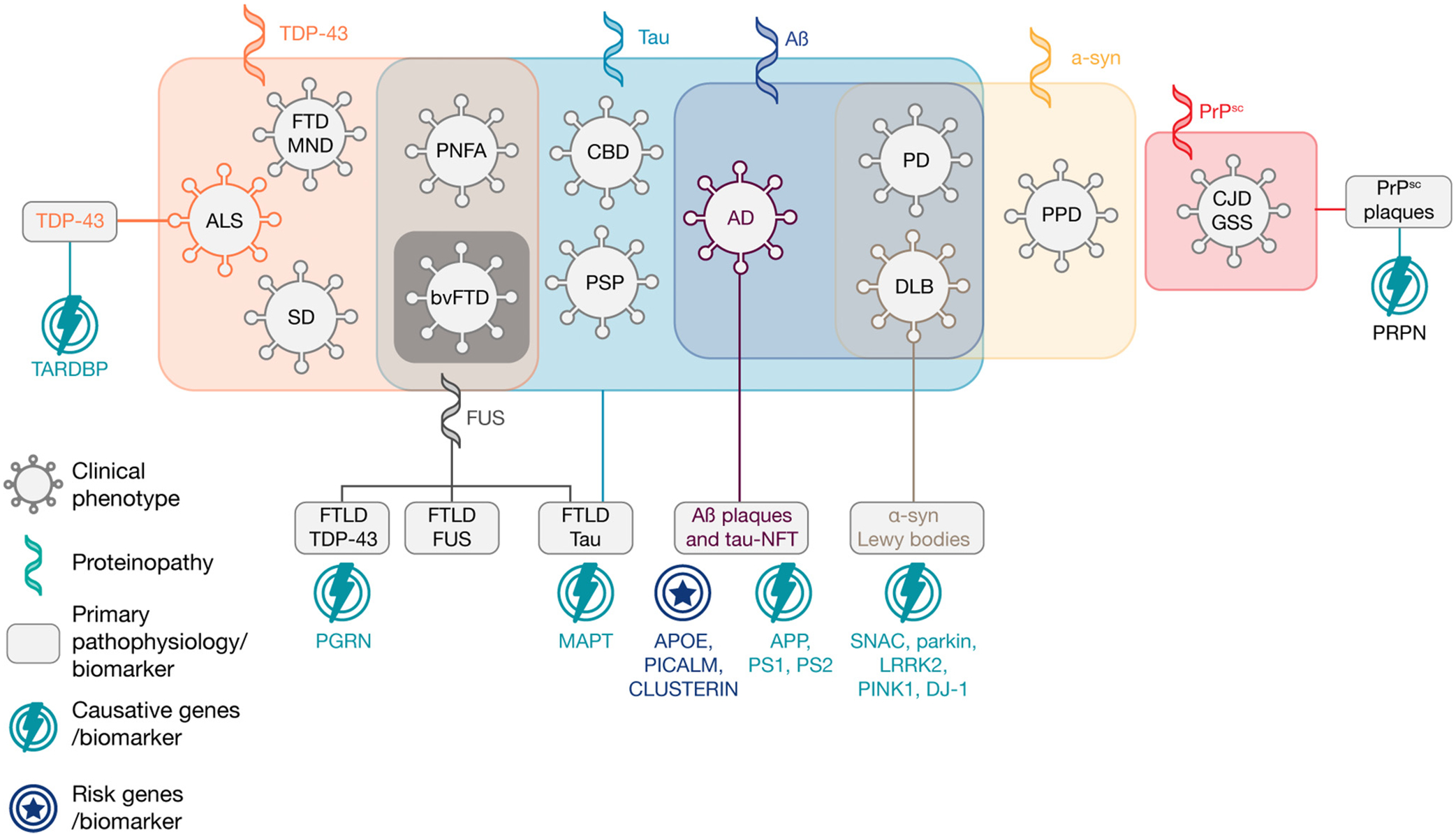
The evolving spectrum of neurodegenerative diseases: from late-stage syndromic phenotypes to extensive genetic–biological–clinical profiling. This schematic describes the evolving, evidence-based concept of neurodegenerative diseases spectrum. Biomarker-guided clinical research showed that conditions with divergent clinical phenotypes exhibit genetic and pathophysiological overlap. By contrast, a traditionally defined clinical phenotype (e.g., behavioral variant of frontotemporal dementia) may have different underlying genetic mutations and pathological alterations, including brain proteinopathies. With comprehensive profiling by the integration of genetic, molecular, and multimodal imaging endophenotypes, current understanding of neurodegenerative diseases continues to evolve and future clinical advances are hoped to overcome the limitations of traditional syndrome-oriented approaches. Figure adapted from the concepts in references [5,31,40]. Abbreviation: Aβ, amyloid β; AD, Alzheimer’s disease; ALS, amyotrophic lateral sclerosis; APOE, apolipoprotein E ε4 allele; APP, amyloid protein precursor; bvFTD, behavioral variant FTD; CBD, corticobasal degeneration; CJD, Creutzfeldt-Jakob disease; DLB, dementia with Lewy bodies; FTD, frontotemporal dementia; FTD-MND, FTD with concurrent motor neuron disease; FTLD, frontotemporal lobar degeneration; FTLD-FUS, FTLD associated with inclusions of protein fused in sarcoma; FTLD-tau, FTLD associated with inclusions of microtubule associated protein tau; FUS, RNA-binding protein FUS; GSS, Gerstmann-Sträussler-Scheinker disease; LRRK2, leucine rich repeat kinase 2; MAPT, microtubule associated protein tau; NFTs, neurofibrillary tangles; PARKIN, parkin RBR E3 ubiquitin-protein ligase; PD, Parkinson’s disease; PGRN, progranulin; PICALM, phosphatidylinositol binding clathrin assembly protein; PINK1, PTEN induced putative kinase 1; PNFA, nonfluent variant primary progressive aphasia; PPD, paranoid personality disorder; PS1, presenilin-1; PS2, presenilin-2; PSP, progressive supranuclear palsy; SD, semantic dementia; SNCA, alpha-synuclein; TDP-43, transactive response DNA-binding protein 43.

Two decades of large-scale observational and systems-scaled studies, including GWAS, have provided insights into pathophysiological pathways of neurodegenerative diseases. These studies revealed that a given syndromic phenotype may be attributable to multiple (epi)genetic and pathological alterations. However, a particular genetic or pathophysiological pattern could also manifest with divergent syndromic phenotypes (Figure 4).

Frameworks for conceptualizing AD have evolved substantially in the past three decades or so. Traditional frameworks focused mostly on syndromic aspects, particularly cognitive decline and progression of functional impairment. Many current perspectives put greater emphasis on clinical–biological constructs, conceived along a continuum, which importantly includes preclinical stages of underlying pathophysiological alterations without overt clinical symptoms [152]. Clinical evidence also increasingly indicates that AD is highly heterogeneous in its susceptibility, risk factors, biological signatures, disease progression, clinical manifestations, and response to treatments [9,36,152]. In addition, sex differences in AD impact disease risk, biomarker profiles, response to treatments, and overall prognosis (Outstanding questions) [153]. Such heterogeneity has complicated clinical studies and partially explains the considerable failure rates of clinical trials [9].

Biomarker profiling offers a key entry point to disentangle disease heterogeneity. In the past two decades, progress has been made in the development of AD fluid and imaging biomarkers. This has led to the conceptualization of a symptom-agnostic, biomarker-based classification system called the Amyloid-β/Tau/Neurodegeneration [AT(N)] system, which stratifies individuals upon core pathophysiological changes in AD [154]. In line with the evolving PM-oriented paradigms, the primary objective of the AT(N) system is to circumvent the limitations of the traditional, clinical phenotype-based approach to AD [152]. As a further step in these developments, the expanding ATX(N) system acts as an extension of the AT(N), where X stands for additional mechanisms (e.g., neuroinflammation and damages to the blood–brain barrier). These biomarker-based classification systems should ultimately inform drug R&D to foster pathway-based, stage-oriented therapeutic strategies in AD. For future clinical practice, the system holds the potential to serve all steps of the evolving AD patient journey from large-scale screening to diagnosis, prognosis, and therapeutic decision-making [62].

Omics sciences studies in AD have already contributed significantly in the quest to decipher the aging–AD continuum, within which upstream genetic polymorphism leads to molecular dynamics accounting for pathomechanistic alterations and downstream biological signatures [9,20,36,53,56,155]. Such an approach has already innovated and boosted AD biomarker/drug target discovery programs [9,20,36,53,56,155]. For example, activated microglia and astrocytes drive and regulate neuroinflammation, an important contributor to AD pathophysiology. Neuroinflammation impacts several finely modulated molecular pathways interacting with other AD pathophysiological pathways (e.g., Aβ and tau), depending on disease stages and individual susceptibility [156,157]. The temporal-spatial dynamics of the neuroinflammatory process could be dissected through multi-omics profiling along with neuroimaging and could potentially be targeted by specific and stage-guided immune-modulator drugs, such as TREM2 agonists, to modify disease progression [157,158].

Consistent with the systems neurophysiology paradigm, multi-modal imaging studies conducted across aging and the AD continuum have pointed to a spatial-temporal overlap of Aβ/tau accumulation with decreased functional connectivity and structural decay in selectively vulnerable regions in large-scale networks, including the default mode network [5,70,112,159]. Such activity and neuroanatomical changes, described initially at the regional level and more recently also in terms of network modular organization, may ultimately allow prediction of long-term cognitive, behavioral, and functional outcomes even in cognitively healthy individuals at risk for AD [5,112,151].

Rapid development in digital health technologies offers an opportunity to detect early signs of AD in a broadly accessible fashion, including the possibility for at-home assessment and monitoring [118,160]. Automated speech analysis is one promising method to detect mild cognitive impairment [161], although clinical validity in this context remains to be further tested. Real-time assessment of eye movement is possible through smart phones/tablets and is being explored as a potential biomarker of early cognitive impairment [162]. Actigraphy recordings provide robust data about motor activity patterns that can be used to infer sleep–awake cycles and other aspects of behaviors (e.g., apathy) in AD patients [163]. Besides screening and diagnosis, digital tools could help quantify and maintain cognitive reserve, which has been linked to resilience against AD and late-life depression [151].

## Challenges and perspectives in clinical research and drug R&D: shared pathophysiological commonalities across diseases

In oncology and clinical immunology, a single compound can exhibit efficacy on a broad set of conditions, for instance various advanced solid tumors, with therapeutic workup guided by profiling specific pathways such as TRK or microsatellite instability-high/DNA mismatch repair (Box 2).

In neurological and psychiatric diseases, a large body of experimental and human evidence points to pathophysiological commonalities involving shared genetic architecture and failure of multiple biological networks, such as proteostasis (e.g., in Aβ and tau pathways), neuronal adaptation and bioenergy regulation, synaptic homeostasis, immune and inflammatory responses [32,38,53]. Using a tactic that has been effective in tumor- and tissue-agnostic cancer therapies, detailed biological profiling of individuals at risk for neurodegenerative diseases, as well as schizoaffective disorders, mood disorders, and ASD, offers an opportunity to develop a new molecular classification system and a related drug R&D program based on distinct biological features and intermediate endophenotypes instead of focusing on syndromic phenotypes (see Outstanding questions) [32,38,53,164].

As part of this conceptual framework, opportunities offered by the emerging field of systems pharmacology should be considered. When standard pharmacodynamic and pharmacokinetic parameters are combined with *in silico* high resolution analyses, systems pharmacological approaches can provide comprehensive information on (epi)genetic regulatory mechanisms of target(s) druggability and drug resistance, as well as simulation of biological pathways down-stream of efficacy and side effects [165,166].

## Accounting for sex-related vulnerability

Large-scale epidemiological observations and multimodal clinical studies indicate the presence of a sex-biased risk to a broad spectrum of neurological and psychiatric diseases [153,167]. Moreover, physiological sexual dimorphism exists in cortical and subcortical structures of the brain, including the limbic system and in grey and white matter connections throughout normal development and diseases [168,169]. In the neurodegenerative spectrum, AD has been extensively investigated to uncover sexual dimorphism across different biological scales [153,167]. For instance, higher vulnerability to AD of menopausal women relative to age-matched men has been linked by cross-disciplinary studies to higher risk of dysregulation of the Aβ and neuroinflammatory pathways, disruptions of the cholinergic nuclei of the basal forebrain, and failure of large-scale networks in the brain [153,170–173]. Such an apparent predisposition of females to AD is not influenced by age itself, thus reinforcing the hypothesis that hormonal factors, some of them linked to menopause, may play a critical role [153,174]. The presence of sexual dimorphism in brain health and disease calls for reconsideration of treatment outcome assessments, taking sex-biased biological factors into account rather than treating sex as a simple covariate [153,175].

## Concluding remarks

Following decades of progress in brain research, and powered by convergent and foundational conceptual-technological breakthroughs, we are now advancing towards the detection of pathophysiological signatures underlying neurological and psychiatric disease at much earlier stages. These advances also allow deconstruction of large, complex, and heterogeneous disease conglomerates into smaller and biologically defined subclusters along the nonlinear dynamic temporal disease continuum. A novel PM approach will rely on biomarker-guided workflows and allow early screening, accurate detection of differentiated pathophysiological signatures, preventative strategies, and time-sensitive, biomarker-guided, pathway-based, targeted therapies tailored to the individual’s specific multidimensional characteristics.

While ambitious in its ultimate aspirations, PM has now arrived at a critical juncture. Neurology has finally entered the intermediate PM development stage, with biomarker-guided pathway-based targeted therapies. The promise of PM for generating mechanistically guided treatments for the suitable patient population, beyond cancer and genetic disorders, has yet to be achieved [8] and examples of ML-powered PM solutions that have significantly impacted clinical practice remain scarce across the spectrum of neuroscience therapeutic areas [144,176]. The PM strategy that has guided recent successes in oncology can inform application and adaptation to neurology and psychiatry. That said, direct accessibility of the affected tissues (and tumors) in living persons for screening and molecular profiling in oncology are not fully transferrable to neurological and psychiatric diseases given the difficulties in direct access to the CNS. In addition, the unique complexity of the anatomical, biological, and genetic architectures of CNS disorders when combined with interindividual heterogeneity can hinder the development of cost-effective biomarkers as a proxy to pathology. For neurological and psychiatric diseases, such strategies need a more sophisticated and differentiated approach to address the underlying systems complexity of brain conditions [177]. Advanced approaches should include integration of rapidly progressing technological areas, such as multi-omics, neuroimaging, neurophysiology, along with clinical and digital phenotype data to accurately subtype CNS diseases and identify druggable targets. As knowledge of human biology and disease pathophysiology advances, it would be possible to perform disease subtyping with increasing granularity. Even so, a balance must be achieved between convergence and divergence of knowledge to ensure that PM can deliver on its inherent potential and help fulfill the promise of improved early patient identification and individualized treatment.

Considering the transformative nature of PM, cross-disciplinary collaboration is essential. To resolve the complex unknowns across CNS disorders, healthcare systems, which are currently clinically operationalized through medical specialties, will require systematic integration of the partially fragmented scientific and medical domains of expertise. Overcoming this barrier also needs enhanced collaboration among stakeholders such as care partners, healthcare providers, regulators, and policy-makers [178]. As a next step, big data science approaches could facilitate the development of these multimodal biomarker variables [50] to support PM-oriented, individualized, stage-dependent treatments for older individuals who suffer from age-related diseases. Ultimately, PM is hoped to offer health span-prolonging solutions throughout different phases of life, such as aging and senescence.

Finally, a PM-oriented approach requires characterization of each individual in the broader context of population-related factors such as sex, ethnicity, geographic location, and socioeconomic status. Just as genetics is influenced by evolutionary dimensions, such as ancestry, environmental and lifestyle factors are impacted by geographic location and socioeconomic status, among other determinants.

One could envision PM-implementation in neurology and psychiatry progress through two major phases. The first phase requires large-scale populations, large enough to include all relevant classifying variables, such as specific genetic and genomic makeup, different ethnicities and sexes, with all the related complex genetic–biological differences, that can then be segmented into subgroups with relatively consistent molecular characteristics and sufficient pathophysiological commonalities, so that each subgroup can be targeted with effective therapeutic and preventive interventions. When harmonized AI-assisted medicine blueprints are increasingly consolidated into clinical research and healthcare practice, PM can transition to its second phase of truly individualized treatments. Achieving these ambitious goals requires first recognizing and embracing human diversity and ensuring inclusion during the different stages of PM development. Hopefully, this path will lead to prolonged health span and better treatments for a wide range of disease conditions, implemented within a broader framework aiming for brain health equity.

## Highlights

Many CNS diseases lack curative or disease-modifying treatments and represent a growing burden to healthcare systems and societies worldwide. These diseases are often multifactorial and complex in nature, with significant individual variability in the underlying genetics and biology.

We posit that the solution to tackling the unmet needs in neurological and psychiatric diseases requires a paradigm shift from a focus on late-stage syndromic phenotypes to targeting preclinical/early prodromal stages.

Precision medicine (PM) approaches in neurology and psychiatry could provide screening solutions, deploy time-sensitive detection/diagnosis, and tailor treatment strategies to an individual’s specific clinical–genetic–biological characteristics and risk factors.

We highlight Alzheimer’s disease as a case in point for PM oriented across neurology and psychiatry and as a compelling model towards PM-oriented drug R&D and healthcare practices.

## Outstanding questions

Mapping clinical–biological trajectories in asymptomatic persons at risk, and in individuals with incipient pathophysiology, is currently challenging. The hurdles stem in part from the complexity of CNS physiology and the simultaneous functional disruption of processes across various hierarchical biological system levels. What could be the ways to validate and standardize systems-level integration approaches in clinical research, based on the PM pillars, to better characterize the underlying genetic–biological nature of CNS diseases?

Integration of multimodal neuroimaging with omics and machine learning-based analysis methods represents a holistic approach to identify groups of individuals with shared genetic architecture and pathophysiology, from molecular pathways up to large-scale networks. Which PM-oriented approaches would be the most effective in drug R&D to create pathway-based, symptoms-agnostic therapies across neurology and psychiatry?

In anticipation to a stage when PM preventive and diagnostic-therapeutic approaches are available for application in neurology and psychiatry, which would be the most relevant implementation strategies to attain healthcare system preparedness?

## Box 1. Precision Medicine Initiatives (PMIs) as a hallmark of an emerging modern era in medicine

The Human Genome Project and the subsequent technological advances in the past few decades have catalyzed rapid progress in human genetics and genomics, yielding new insights into the biological basis of a wide range of health and disease states [2,20]. Recent progress in omics (genomics, transcriptomics, epigenomics, proteomics, lipidomics, metabolomics) and related clinical research applications hold the potential for comprehensive molecular profiling of complex diseases to track their biological evolution across spatial and temporal scales and through different clinical stages. In this context, systems theory as the study of systems composed of interrelated, interdependent parts and its applied sciences (systems biology, systems neurophysiology, and quantitative systems pharmacology) provide a conceptual and analytical framework to generate explainable and biologically clinically meaningful readouts. Large and multidimensional datasets are increasingly available for single or multi-omics studies; several global working groups and societies have formed to accelerate the translation of omics signatures to pharmacological research and/or clinical practice [18,19,179,180].

There is rapid growth of enriched and systematized clinical data in electronic health records (EHRs) and other health-related information databases via digital technologies, such as wearable devices, smartphones, and edge computing [181]. In parallel, tremendous growth and maturation of computational science has stimulated the field of bioinformatics and applied artificial intelligence (AI) in the past decade. These converging theoretical and scientific advances have catalyzed the conceptual and technical foundation for PM as a core element of a new era of medicine [178].

In 2015, the PMI was launched in the USA with a near-term focus on oncology [1]. Its long-term vision is to generate knowledge across health and disease that will ultimately enable a more complete understanding of disease mechanisms, better assessment of disease risk, and improved prediction of optimal therapy [1]. A key element of the PMI is the ‘All of Us’ research program, which aims to collect, including from ethnically diverse individuals in the USA, biospecimens, physical measurements, and other health-related information and link these to EHRs with plans to follow the participants longitudinally for decades [182]. The large-scale ‘All of Us’ clinical research program offers unprecedented opportunities to investigate a broad medical spectrum and identify health outcomes for integrative and holistic evaluation, accurate diagnosis, development of biomarker-guided targeted therapies of diverse subsets of individuals, treatment selection, real-world and outcome research, and evidence-based prevention [182]. A similar initiative in the UK, the UK Biobank, has created a robust biomedical database that can be accessed globally by different stakeholders for clinical and public health research [183]. With whole-genome sequencing, neuroimaging, and extensive medical characterization for over half a million UK residents, the UK Biobank has become a platform to discover primary factors that affect short- and long-term health outcomes. This success has stimulated parallel developments in national biobanks worldwide and efforts to harmonize, merge, and compare biobank data across countries and continents [19].

## Box 2. Precision oncology

The field of oncology has pioneered the development and implementation of the PM-oriented holistic and patient-centered approach to research and clinical care [23]. Today, PM is being applied throughout the oncology clinical care spectrum, from risk assessment to screening, detection, diagnosis, staging and prognosis, therapy selection, and monitoring.

PM aims to change expectations and behavior toward prevention and prolonging health rather than focusing on diagnostic work-up and therapeutic intervention only when the disease has manifested clinically. Screening represents the most effective strategy for risk prediction and early detection/disease prevention [182]. Noninvasive and globally accessible screening tests that measure the genetic and/or biological variation and risks have been developed, validated, and qualified and are widely available for various types of cancer [184].

In addition to screening, the field of oncology routinely applies genomic and other biomarker analyses on tumor samples and, increasingly, also on bodily fluid samples such as blood (i.e., liquid biopsy) [185], to guide diagnostic and therapeutic decision-making [186]. Moreover, progress in the integration of AI with traditional pathology- or biomarker-based diagnostic work-up holds the promise to address some of the key medical challenges, such as missed or delayed diagnosis and limited resources of healthcare systems [187].

Finally, oncology is a most suitable testing ground for PM-oriented drug R&D, as cancer is a highly heterogeneous biological condition with diverse molecular underpinnings and effective treatments need to target the genomic and other molecular characteristics of patients and their tumors. Drug development under this framework has led to the approval of several tumor- and site-agnostic treatments. In 2017, the US FDA approved pembrolizumab as an immunotherapy treatment for cancers expressing anti-programmed cell death protein 1 regardless of the clinical manifestation such as tumor site or histology. Pembrolizumab indications span a broad set of advanced solid tumors, with a therapeutic work-up guided by the investigation of microsatellite instability-high/DNA mismatch repair-deficient biomarkers rather than clinical phenotypes [24]. Other compounds are in development programs aligned with this approach, such as larotrectinib for TRK fusion-positive cancers [188]. Importantly, these drug development approaches are based on the molecular/biological characteristics of the tumor rather than the overt clinical manifestations such as tumor type/site, increasing the overall clinical benefit and avoiding unnecessary toxicity.

## Box 3. Liquid biopsy

Minimally invasive and globally accessible tests for different contexts-of-use are urgently needed to address the growing demand for timely diagnosis and management of neurological and psychiatric disease. Blood-based biomarkers are cost-, resource-, and time effective [67]. They hold the potential to enable large-scale biological screening to identify individuals who are likely to have disease-specific pathophysiology and to determine the need for second-level, less accessible or more invasive investigations (e.g., PET or CSF assessment) [64]. Blood-based biomarkers can provide the opportunity for a more efficient, multistep diagnostic work-up. Further, they can facilitate the re-engineering of drug R&D pipelines, from subject enrollment, target engagement, to monitoring of treatment efficacy.

The oncology field has pioneered the approach of liquid biopsy, a concept which could cross-fertilize related practices in neurology and psychiatry programs [185]. According to the US National Cancer Institute, liquid biopsy is defined as ‘a test done on a sample of blood to look for cancer cells from a tumor that are circulating in the blood or for pieces of DNA from tumor cells that are in the blood’. Liquid biopsy in oncology allows the detection of various tumor-specific circulating analytes (circulating cell-free DNA and RNA, circulating tumor DNA, extracellular vesicles, etc.) that carry information about the genome/epigenome, transcriptome, proteome, and metabolome of the tumor. Then, these multidimensional big data are integrated through advanced bioinformatics to detect molecular signatures relevant to the disease pathophysiology. Serial liquid biopsies offer clues about the evolution of cancer in individual patients across disease stages, enabling individualized, genetically and biologically guided therapies [185]. In translating such an approach to CNS diseases, the development of novel ultrasensitive, high-throughput techniques has enabled the detection of multidimensional fluid biomarkers that circumvent neuroanatomical barriers and provide comprehensive snapshots of brain pathophysiological processes [65].

## References

[1] Collins FS and Varmus H (2015) A new initiative on precision medicine. N. Engl. J. Med 372, 793–795 25635347 10.1056/NEJMp1500523 PMC5101938

[2] Green ED (2020) Strategic vision for improving human health at the forefront of genomics. Nature 586, 683–692 33116284 10.1038/s41586-020-2817-4 PMC7869889

[3] Hess JL (2021) A polygenic resilience score moderates the genetic risk for schizophrenia. Mol. Psychiatry 26, 800–815 31492941 10.1038/s41380-019-0463-8 PMC7058518

[4] Kesselheim AS (2015) Two decades of new drug development for central nervous system disorders. Nat. Rev. Drug Discov 14, 815–816 26585536 10.1038/nrd4793

[5] Yu M (2021) The human connectome in Alzheimer disease - relationship to biomarkers and genetics. Nat. Rev. Neurol 17, 545–563 34285392 10.1038/s41582-021-00529-1 PMC8403643

[6] Vigo D (2016) Estimating the true global burden of mental illness. Lancet Psychiatry 3, 171–178 26851330 10.1016/S2215-0366(15)00505-2

[7] GBD (2016) Neurology Collaborators (2019) Global, regional, and national burden of neurological disorders, 1990–2016: a systematic analysis for the Global Burden of Disease Study 2016. Lancet Neurol. 18, 459–480 10.1016/S1474-4422(18)30499-X PMC6459001 30879893

[8] Dugger SA (2018) Drug development in the era of precision medicine. Nat. Rev. Drug Discov 17, 183–196 29217837 10.1038/nrd.2017.226 PMC6287751

[9] Karran E and Hardy J (2014) A critique of the drug discovery and phase 3 clinical programs targeting the amyloid hypothesis for Alzheimer disease. Ann. Neurol 76, 185–205 24853080 10.1002/ana.24188 PMC4204160

[10] Noori A (2021) Systematic review and meta-analysis of human transcriptomics reveals neuroinflammation, deficient energy metabolism, and proteostasis failure across neurodegeneration. Neurobiol. Dis 149, 105225 33347974 10.1016/j.nbd.2020.105225 PMC7856076

[11] Chang MC (2020) The effectiveness of nonsteroidal anti-inflammatory drugs and acetaminophen in reduce the risk of amyotrophic lateral sclerosis? A meta-analysis. Sci. Rep 10, 14759 32901053 10.1038/s41598-020-71813-1 PMC7479139

[12] Becker RE and Greig NH (2010) Lost in translation: neuropsychiatric drug development. Sci. Transl. Med 2, 61rv66 10.1126/scitranslmed.3000446 PMC5178822 21148128

[13] Perez-Nievas BG (2013) Dissecting phenotypic traits linked to human resilience to Alzheimer’s pathology. Brain 136, 2510–2526 23824488 10.1093/brain/awt171 PMC3722351

[14] Elman JA (2014) Neural compensation in older people with brain amyloid-beta deposition. Nat. Neurosci 17, 1316–1318 25217827 10.1038/nn.3806 PMC4177011

[15] Gomez-Isla T and Frosch MP (2022) Lesions without symptoms: understanding resilience to Alzheimer disease neuropathological changes. Nat. Rev. Neurol 18, 323–332 35332316 10.1038/s41582-022-00642-9 PMC10607925

[16] Nedergaard M and Goldman SA (2020) Glymphatic failure as a final common pathway to dementia. Science 370, 50–56 33004510 10.1126/science.abb8739 PMC8186542

[17] Selkoe DJ (2002) Alzheimer’s disease is a synaptic failure. Science 298, 789–791 12399581 10.1126/science.1074069

[18] Schwarz E (2021) Identifying multimodal signatures underlying the somatic comorbidity of psychosis: the COMMITMENT roadmap. Mol. Psychiatry 26, 722–724 33060817 10.1038/s41380-020-00915-z PMC7910206

[19] Thompson PM (2020) ENIGMA and global neuroscience: a decade of large-scale studies of the brain in health and disease across more than 40 countries. Transl. Psychiatry 10, 100 32198361 10.1038/s41398-020-0705-1 PMC7083923

[20] Hampel H (2019) Time for the systems-level integration of aging: resilience enhancing strategies to prevent Alzheimer’s disease. Prog. Neurobiol 181, 101662 31351912 10.1016/j.pneurobio.2019.101662

[21] Hood L and Friend SH (2011) Predictive, personalized, preventive, participatory (P4) cancer medicine. Nat. Rev. Clin. Oncol 8, 184–187 21364692 10.1038/nrclinonc.2010.227

[22] Nabbout R and Kuchenbuch M (2020) Impact of predictive, preventive and precision medicine strategies in epilepsy. Nat. Rev. Neurol 16, 674–688 33077944 10.1038/s41582-020-0409-4

[23] Prasad V (2016) Precision oncology: origins, optimism, and potential. Lancet Oncol. 17, e81–e86 26868357 10.1016/S1470-2045(15)00620-8

[24] Goldberg KB (2018) The FDA Oncology Center of Excellence and precision medicine. Exp. Biol. Med. (Maywood) 243, 308–312 29105511 10.1177/1535370217740861 PMC5813869

[25] Yan L and Zhang W (2018) Precision medicine becomes reality-tumor type-agnostic therapy. Cancer Commun. (Lond.) 38, 6 29764494 10.1186/s40880-018-0274-3 PMC5953403

[26] Mercuri E (2020) Spinal muscular atrophy - insights and challenges in the treatment era. Nat. Rev. Neurol 16, 706–715 33057172 10.1038/s41582-020-00413-4

[27] Neuner SM (2020) Genetic architecture of Alzheimer’s disease. Neurobiol. Dis 143, 104976 32565066 10.1016/j.nbd.2020.104976 PMC7409822

[28] Blauwendraat C (2020) The genetic architecture of Parkinson’s disease. Lancet Neurol. 19, 170–178 31521533 10.1016/S1474-4422(19)30287-X PMC8972299

[29] Tabrizi SJ (2020) Huntington disease: new insights into molecular pathogenesis and therapeutic opportunities. Nat. Rev. Neurol 16, 529–546 32796930 10.1038/s41582-020-0389-4

[30] Gejman PV (2011) Genetics of schizophrenia: new findings and challenges. Annu. Rev. Genomics Hum. Genet 12, 121–144 21639796 10.1146/annurev-genom-082410-101459

[31] Vorstman JAS (2017) Autism genetics: opportunities and challenges for clinical translation. Nat. Rev. Genet 18, 362–376 28260791 10.1038/nrg.2017.4

[32] Sanders SJ (2019) A framework for the investigation of rare genetic disorders in neuropsychiatry. Nat. Med 25, 1477–1487 31548702 10.1038/s41591-019-0581-5 PMC8656349

[33] Geschwind DH and Flint J (2015) Genetics and genomics of psychiatric disease. Science 349, 1489–1494 26404826 10.1126/science.aaa8954 PMC4694563

[34] Schizophrenia Working Group of the Psychiatric Genomics Consortium (2014) Biological insights from 108 schizophrenia-associated genetic loci. Nature 511, 421–427 25056061 10.1038/nature13595 PMC4112379

[35] Schwartzentruber J (2021) Genome-wide meta-analysis, fine-mapping and integrative prioritization implicate new Alzheimer’s disease risk genes. Nat. Genet 53, 392–402 33589840 10.1038/s41588-020-00776-w PMC7610386

[36] de Rojas I (2021) Common variants in Alzheimer’s disease and risk stratification by polygenic risk scores. Nat. Commun 12, 3417 34099642 10.1038/s41467-021-22491-8 PMC8184987

[37] Nalls MA (2014) Large-scale meta-analysis of genome-wide association data identifies six new risk loci for Parkinson’s disease. Nat. Genet 46, 989–993 25064009 10.1038/ng.3043 PMC4146673

[38] De Jager PL (2018) Deconstructing and targeting the genomic architecture of human neurodegeneration. Nat. Neurosci 21, 1310–1317 30258235 10.1038/s41593-018-0240-z

[39] McCarroll SA and Hyman SE (2013) Progress in the genetics of polygenic brain disorders: significant new challenges for neurobiology. Neuron 80, 578–587 24183011 10.1016/j.neuron.2013.10.046 PMC4066986

[40] Lewis CM and Vassos E (2020) Polygenic risk scores: from research tools to clinical instruments. Genome Med. 12, 44 32423490 10.1186/s13073-020-00742-5 PMC7236300

[41] Chasioti D (2019) Progress in polygenic composite scores in Alzheimer’s and other complex diseases. Trends Genet. 35, 371–382 30922659 10.1016/j.tig.2019.02.005 PMC6475476

[42] Jonas KG (2019) Schizophrenia polygenic risk score and 20-year course of illness in psychotic disorders. Transl. Psychiatry 9, 300 31727878 10.1038/s41398-019-0612-5 PMC6856168

[43] Serdarevic F (2020) Polygenic risk scores for developmental disorders, neuromotor functioning during infancy, and autistic traits in childhood. Biol. Psychiatry 87, 132–138 31629460 10.1016/j.biopsych.2019.06.006

[44] Chaudhury S (2019) Alzheimer’s disease polygenic risk score as a predictor of conversion from mild-cognitive impairment. Transl. Psychiatry 9, 154 31127079 10.1038/s41398-019-0485-7 PMC6534556

[45] Leonenko G (2021) Identifying individuals with high risk of Alzheimer’s disease using polygenic risk scores. Nat. Commun 12, 4506 34301930 10.1038/s41467-021-24082-z PMC8302739

[46] Rampino A (2017) A polygenic risk score of glutamatergic SNPs associated with schizophrenia predicts attentional behavior and related brain activity in healthy humans. Eur. Neuropsychopharmacol 27, 928–939 28651857 10.1016/j.euroneuro.2017.06.005

[47] Ranlund S (2018) A polygenic risk score analysis of psychosis endophenotypes across brain functional, structural, and cognitive domains. Am. J. Med. Genet. B Neuropsychiatr. Genet 177, 21–34 28851104 10.1002/ajmg.b.32581 PMC5763362

[48] Torkamani A (2018) The personal and clinical utility of polygenic risk scores. Nat. Rev. Genet 19, 581–590 29789686 10.1038/s41576-018-0018-x

[49] Chen R (2012) Personal omics profiling reveals dynamic molecular and medical phenotypes. Cell 148, 1293–1307 22424236 10.1016/j.cell.2012.02.009 PMC3341616

[50] Schussler-Fiorenza Rose SM (2019) A longitudinal big data approach for precision health. Nat. Med 25, 792–804 31068711 10.1038/s41591-019-0414-6 PMC6713274

[51] Garrett-Bakelman FE (2019) The NASA Twins Study: a multidimensional analysis of a year-long human spaceflight. Science 364, eaau8650 30975860 10.1126/science.aau8650 PMC7580864

[52] Klein HU (2016) The epigenome in Alzheimer’s disease: current state and approaches for a new path to gene discovery and understanding disease mechanism. Acta Neuropathol. 132, 503–514 27573688 10.1007/s00401-016-1612-7 PMC5189639

[53] Hampel H (2021) Omics sciences for systems biology in Alzheimer’s disease: state-of-the-art of the evidence. Ageing Res. Rev 69, 101346 33915266 10.1016/j.arr.2021.101346

[54] Borrageiro G (2018) A review of genome-wide transcriptomics studies in Parkinson’s disease. Eur. J. Neurosci 47, 1–16 29068110 10.1111/ejn.13760

[55] Hernandez LM (2021) Transcriptomic insight into the polygenic mechanisms underlying psychiatric disorders. Biol. Psychiatry 89, 54–64 32792264 10.1016/j.biopsych.2020.06.005 PMC7718368

[56] Johnson ECB (2020) Large-scale proteomic analysis of Alzheimer’s disease brain and cerebrospinal fluid reveals early changes in energy metabolism associated with microglia and astrocyte activation. Nat. Med 26, 769–780 32284590 10.1038/s41591-020-0815-6 PMC7405761

[57] Ping L (2020) Global quantitative analysis of the human brain proteome and phosphoproteome in Alzheimer’s disease. Sci. Data 7, 315 32985496 10.1038/s41597-020-00650-8 PMC7522715

[58] Notaras M (2021) The proteomic architecture of schizophrenia iPSC-derived cerebral organoids reveals alterations in GWAS and neuronal development factors. Transl. Psychiatry 11, 541 34667143 10.1038/s41398-021-01664-5 PMC8526592

[59] Glinton KE and Elsea SH (2019) Untargeted metabolomics for autism spectrum disorders: current status and future directions. Front. Psychiatry 10, 647 31551836 10.3389/fpsyt.2019.00647 PMC6746843

[60] Varma VR (2018) Brain and blood metabolite signatures of pathology and progression in Alzheimer disease: a targeted metabolomics study. PLoS Med. 15, e1002482 29370177 10.1371/journal.pmed.1002482 PMC5784884

[61] Shao Y and Le W (2019) Recent advances and perspectives of metabolomics-based investigations in Parkinson’s disease. Mol. Neurodegener 14, 3 30634989 10.1186/s13024-018-0304-2 PMC6330496

[62] Hampel H (2021) Developing the ATX(N) classification for use across the Alzheimer disease continuum. Nat. Rev. Neurol 17, 580–589 34239130 10.1038/s41582-021-00520-w

[63] Andersen AD (2017) Cerebrospinal fluid biomarkers for Parkinson’s disease - a systematic review. Acta Neurol. Scand 135, 34–56 26991855 10.1111/ane.12590

[64] Molinuevo JL (2018) Current state of Alzheimer’s fluid biomarkers. Acta Neuropathol. 136, 821–853 30488277 10.1007/s00401-018-1932-x PMC6280827

[65] Hampel H (2019) Biomarker-drug and liquid biopsy co-development for disease staging and targeted therapy: corner-stones for Alzheimer’s precision medicine and pharmacology. Front. Pharmacol 10, 310 30984002 10.3389/fphar.2019.00310 PMC6450260

[66] Teunissen CE (2021) Blood-based biomarkers for Alzheimer’s disease: towards clinical implementation. Lancet Neurol. 21, 66–77 34838239 10.1016/S1474-4422(21)00361-6

[67] Hampel H (2018) Blood-based biomarkers for Alzheimer disease: mapping the road to the clinic. Nat. Rev. Neurol 14, 639–652 30297701 10.1038/s41582-018-0079-7 PMC6211654

[68] Parnetti L (2019) CSF and blood biomarkers for Parkinson’s disease. Lancet Neurol. 18, 573–586 30981640 10.1016/S1474-4422(19)30024-9

[69] Levin F (2021) In vivo staging of regional amyloid progression in healthy middle-aged to older people at risk of Alzheimer’s disease. Alzheimers Res. Ther 13, 178 34674764 10.1186/s13195-021-00918-0 PMC8532333

[70] Villemagne VL (2018) Imaging tau and amyloid-beta proteinopathies in Alzheimer disease and other conditions. Nat. Rev. Neurol 14, 225–236 29449700 10.1038/nrneurol.2018.9

[71] Mullin S (2021) Brain microglial activation increased in glucocerebrosidase (GBA) mutation carriers without Parkinson’s disease. Mov. Disord 36, 774–779 33278043 10.1002/mds.28375 PMC8048428

[72] Cumming P (2021) Molecular imaging of schizophrenia: neurochemical findings in a heterogeneous and evolving disorder. Behav. Brain Res 398, 113004 33197459 10.1016/j.bbr.2020.113004

[73] McKeith IG (2020) Research criteria for the diagnosis of prodromal dementia with Lewy bodies. Neurology 94, 743–755 32241955 10.1212/WNL.0000000000009323 PMC7274845

[74] Postuma RB (2015) MDS clinical diagnostic criteria for Parkinson’s disease. Mov. Disord 30, 1591–1601 26474316 10.1002/mds.26424

[75] Valizadeh SA (2017) Age prediction on the basis of brain anatomical measures. Hum. Brain Mapp 38, 997–1008 27807912 10.1002/hbm.23434 PMC6866800

[76] Valizadeh SA (2018) Identification of individual subjects on the basis of their brain anatomical features. Sci. Rep 8, 5611 29618790 10.1038/s41598-018-23696-6 PMC5884835

[77] Finc K (2020) Dynamic reconfiguration of functional brain networks during working memory training. Nat. Commun 11, 2435 32415206 10.1038/s41467-020-15631-z PMC7229188

[78] Drysdale AT (2017) Resting-state connectivity biomarkers define neurophysiological subtypes of depression. Nat. Med 23, 28–38 27918562 10.1038/nm.4246 PMC5624035

[79] Whitfield-Gabrieli S (2016) Brain connectomics predict response to treatment in social anxiety disorder. Mol. Psychiatry 21, 680–685 26260493 10.1038/mp.2015.109

[80] Yahata N (2016) A small number of abnormal brain connections predicts adult autism spectrum disorder. Nat. Commun 7, 11254 27075704 10.1038/ncomms11254 PMC4834637

[81] Mash LE (2020) Atypical relationships between spontaneous EEG and fMRI activity in autism. Brain Connect 10, 18–28 31884804 10.1089/brain.2019.0693 PMC7044766

[82] Gawne TJ (2020) A multimodal magnetoencephalography 7 T fMRI and 7 T proton MR spectroscopy study in first episode psychosis. NPJ Schizophr. 6, 23 32887887 10.1038/s41537-020-00113-4 PMC7473853

[83] Sui J (2014) Combination of FMRI-SMRI-EEG data improves discrimination of schizophrenia patients by ensemble feature selection. Annu. Int. Conf. IEEE Eng. Med. Biol. Soc 2014, 3889–3892 25570841 10.1109/EMBC.2014.6944473

[84] Ford JM (2016) Using concurrent EEG and fMRI to probe the state of the brain in schizophrenia. Neuroimage Clin. 12, 429–441 27622140 10.1016/j.nicl.2016.08.009 PMC5008052

[85] Smit DJA (2018) Genome-wide association analysis links multiple psychiatric liability genes to oscillatory brain activity. Hum. Brain Mapp 39, 4183–4195 29947131 10.1002/hbm.24238 PMC6179948

[86] Barabasi AL (2011) Network medicine: a network-based approach to human disease. Nat. Rev. Genet 12, 56–68 21164525 10.1038/nrg2918 PMC3140052

[87] Kuenzi BM and Ideker T (2020) A census of pathway maps in cancer systems biology. Nat. Rev. Cancer 20, 233–246 32066900 10.1038/s41568-020-0240-7 PMC7224610

[88] Maron BA (2021) Individualized interactomes for network-based precision medicine in hypertrophic cardiomyopathy with implications for other clinical pathophenotypes. Nat. Commun 12, 873 33558530 10.1038/s41467-021-21146-y PMC7870822

[89] Bullmore E and Sporns O (2009) Complex brain networks: graph theoretical analysis of structural and functional systems. Nat. Rev. Neurosci 10, 186–198 19190637 10.1038/nrn2575

[90] Hood L (2004) Systems biology and new technologies enable predictive and preventative medicine. Science 306, 640–643 15499008 10.1126/science.1104635

[91] Radulescu E (2020) Identification and prioritization of gene sets associated with schizophrenia risk by co-expression network analysis in human brain. Mol. Psychiatry 25, 791–804 30478419 10.1038/s41380-018-0304-1

[92] Hu YS (2017) Analyzing the genes related to Alzheimer’s disease via a network and pathway-based approach. Alzheimers Res. Ther 9, 29 28446202 10.1186/s13195-017-0252-z PMC5406904

[93] Okuda S (2008) KEGG Atlas mapping for global analysis of metabolic pathways. Nucleic Acids Res. 36, W423–W426 18477636 10.1093/nar/gkn282 PMC2447737

[94] Yan J (2018) Network approaches to systems biology analysis of complex disease: integrative methods for multiomics data. Brief. Bioinform 19, 1370–1381 28679163 10.1093/bib/bbx066 PMC6454489

[95] Wang M (2021) Transformative network modeling of multi-omics data reveals detailed circuits, key regulators, and potential therapeutics for Alzheimer’s disease. Neuron 109, 257–272 33238137 10.1016/j.neuron.2020.11.002 PMC7855384

[96] Wang Q (2019) A Bayesian framework that integrates multi-omics data and gene networks predicts risk genes from schizophrenia GWAS data. Nat. Neurosci 22, 691–699 30988527 10.1038/s41593-019-0382-7 PMC6646046

[97] Wang D (2018) Comprehensive functional genomic resource and integrative model for the human brain. Science 362, eaat8464 30545857 10.1126/science.aat8464 PMC6413328

[98] Hampel H (2018) Revolution of Alzheimer precision neurology. Passageway of systems biology and neurophysiology. J. Alzheimers Dis 64, S47–S105 29562524 10.3233/JAD-179932 PMC6008221

[99] Harrison TM (2019) Longitudinal tau accumulation and atrophy in aging and alzheimer disease. Ann. Neurol 85, 229–240 30597624 10.1002/ana.25406 PMC6579738

[100] Scott MR (2020) Inferior temporal tau is associated with accelerated prospective cortical thinning in clinically normal older adults. Neuroimage 220, 116991 32512123 10.1016/j.neuroimage.2020.116991 PMC7572623

[101] Drzezga A (2011) Neuronal dysfunction and disconnection of cortical hubs in non-demented subjects with elevated amyloid burden. Brain 134, 1635–1646 21490054 10.1093/brain/awr066 PMC3102239

[102] Harrison TM (2021) Distinct effects of beta-amyloid and tau on cortical thickness in cognitively healthy older adults. Alzheimers Dement. 17, 1085–1096 33325068 10.1002/alz.12249 PMC8203764

[103] Brueggen K (2017) Early changes in alpha band power and DMN BOLD activity in Alzheimer’s disease: a simultaneous resting state EEG-fMRI study. Front. Aging Neurosci 9, 319 29056904 10.3389/fnagi.2017.00319 PMC5635054

[104] Franzmeier N (2020) Functional brain architecture is associated with the rate of tau accumulation in Alzheimer’s disease. Nat. Commun 11, 347 31953405 10.1038/s41467-019-14159-1 PMC6969065

[105] Liu C (2020) Brain functional and structural signatures in Parkinson’s disease. Front. Aging Neurosci 12, 125 32528272 10.3389/fnagi.2020.00125 PMC7264099

[106] Adams RA (2020) Impaired theta phase coupling underlies frontotemporal dysconnectivity in schizophrenia. Brain 143, 1261–1277 32236540 10.1093/brain/awaa035 PMC7174039

[107] Syed Nasser N (2019) Incremental benefits of EEG informed fMRI in the study of disorders related to meso-corticolimbic dopamine pathway dysfunction: a systematic review of recent literature. J. Clin. Neurosci 65, 87–99 30955950 10.1016/j.jocn.2019.03.054

[108] Sommerauer M (2018) Evaluation of the noradrenergic system in Parkinson’s disease: an 11C-MeNER PET and neuromelanin MRI study. Brain 141, 496–504 29272343 10.1093/brain/awx348

[109] Chung JW (2018) Beta-band oscillations in the supplementary motor cortex are modulated by levodopa and associated with functional activity in the basal ganglia. Neuroimage Clin. 19, 559–571 29984164 10.1016/j.nicl.2018.05.021 PMC6029579

[110] Smailovic U (2020) Regional disconnection in Alzheimer dementia and amyloid-positive mild cognitive impairment: association between EEG functional connectivity and brain glucose metabolism. Brain Connect 10, 555–565 33073602 10.1089/brain.2020.0785 PMC7757561

[111] Sanchez-Catasus CA (2021) Dopaminergic nigrostriatal connectivity in early Parkinson disease: in vivo neuroimaging study of (11)C-DTBZ PET combined with correlational tractography. J. Nucl. Med 62, 545–552 32859707 10.2967/jnumed.120.248500 PMC8973250

[112] Babiloni C (2020) Resting-state posterior alpha rhythms are abnormal in subjective memory complaint seniors with preclinical Alzheimer’s neuropathology and high education level: the INSIGHT-preAD study. Neurobiol. Aging 90, 43–59 32111391 10.1016/j.neurobiolaging.2020.01.012

[113] Babiloni C (2016) Cortical sources of resting state EEG rhythms are related to brain hypometabolism in subjects with Alzheimer’s disease: an EEG-PET study. Neurobiol. Aging 48, 122–134 27668356 10.1016/j.neurobiolaging.2016.08.021

[114] Hansen JY (2021) Mapping neurotransmitter systems to the structural and functional organization of the human neocortex. Nat. Neurosci 25, 1569–1581 10.1038/s41593-022-01186-3 PMC9630096 36303070

[115] Writing Committee for the Attention-Deficit/Hyperactivity Disorder (2021) Virtual histology of cortical thickness and shared neurobiology in 6 psychiatric disorders. JAMA Psychiatry 78, 47–63 32857118 10.1001/jamapsychiatry.2020.2694 PMC7450410

[116] Rubinov M and Sporns O (2010) Complex network measures of brain connectivity: uses and interpretations. Neuroimage 52, 1059–1069 19819337 10.1016/j.neuroimage.2009.10.003

[117] Lariviere S (2020) Network-based atrophy modeling in the common epilepsies: a worldwide ENIGMA study. Sci. Adv 6, eabc6457 33208365 10.1126/sciadv.abc6457 PMC7673818

[118] Kourtis LC (2019) Digital biomarkers for Alzheimer’s disease: the mobile/ wearable devices opportunity. NPJ Digit. Med 2, 9 31119198 10.1038/s41746-019-0084-2 PMC6526279

[119] Lipsmeier F (2018) Evaluation of smartphone-based testing to generate exploratory outcome measures in a phase 1 Parkinson’s disease clinical trial. Mov. Disord 33, 1287–1297 29701258 10.1002/mds.27376 PMC6175318

[120] Artusi CA (2018) Integration of technology-based outcome measures in clinical trials of Parkinson and other neurodegenerative diseases. Parkinsonism Relat. Disord 46, S53–S56 28760593 10.1016/j.parkreldis.2017.07.022 PMC7924909

[121] Espay AJ (2019) A roadmap for implementation of patient-centered digital outcome measures in Parkinson’s disease obtained using mobile health technologies. Mov. Disord 34, 657–663 30901495 10.1002/mds.27671 PMC6520192

[122] Tortelli R (2021) The use of wearable/portable digital sensors in Huntington’s disease: a systematic review. Parkinsonism Relat. Disord 83, 93–104 33493786 10.1016/j.parkreldis.2021.01.006 PMC7957324

[123] Zhan A (2018) Using smartphones and machine learning to quantify Parkinson disease severity: the mobile Parkinson disease score. JAMA Neurol. 75, 876–880 29582075 10.1001/jamaneurol.2018.0809 PMC5885192

[124] Powers R (2021) Smartwatch inertial sensors continuously monitor real-world motor fluctuations in Parkinson’s disease. Sci. Transl. Med 13, eabd7865 33536284 10.1126/scitranslmed.abd7865

[125] Zulueta J (2018) Predicting mood disturbance severity with mobile phone keystroke metadata: a BiAffect digital phenotyping study. J. Med. Internet Res 20, e241 30030209 10.2196/jmir.9775 PMC6076371

[126] Mohr DC (2018) A solution-focused research approach to achieve an implementable revolution in digital mental health. JAMA Psychiatry 75, 113–114 29238805 10.1001/jamapsychiatry.2017.3838

[127] Canazei M (2019) Actigraphy for assessing light effects on sleep and circadian activity rhythm in Alzheimer’s dementia: a narrative review. Curr. Alzheimer Res 16, 1084–1107 31608835 10.2174/1567205016666191010124011

[128] Barrett PM (2017) Digitising the mind. Lancet 389, 1877 28513442 10.1016/S0140-6736(17)31218-7

[129] Firth J (2017) The efficacy of smartphone-based mental health interventions for depressive symptoms: a meta-analysis of randomized controlled trials. World Psychiatry 16, 287–298 28941113 10.1002/wps.20472 PMC5608852

[130] Myszczynska MA (2020) Applications of machine learning to diagnosis and treatment of neurodegenerative diseases. Nat. Rev. Neurol 16, 440–456 32669685 10.1038/s41582-020-0377-8

[131] Jordan MI and Mitchell TM (2015) Machine learning: trends, perspectives, and prospects. Science 349, 255–260 26185243 10.1126/science.aaa8415

[132] Lu BL (2022) A practical Alzheimer disease classifier via brain imaging-based deep learning on 85,721 samples. J. Big Data 9, 101

[133] Wang T (2021) A review on medical imaging synthesis using deep learning and its clinical applications. J. Appl. Clin. Med. Phys 22, 11–36 10.1002/acm2.13121 PMC7856512 33305538

[134] Chen YH (2018) Brain MRI super resolution using 3D deep densely connected neural networks. In 2018 IEEE 15th International Symposium on Biomedical Imaging, IEEE

[135] Richards BA (2019) A deep learning framework for neuroscience. Nat. Neurosci 22, 1761–1770 31659335 10.1038/s41593-019-0520-2 PMC7115933

[136] Schnack HG (2019) Improving individual predictions: machine learning approaches for detecting and attacking heterogeneity in schizophrenia (and other psychiatric diseases). Schizophr. Res 214, 34–42 29074332 10.1016/j.schres.2017.10.023

[137] Rathore S (2017) A review on neuroimaging-based classification studies and associated feature extraction methods for Alzheimer’s disease and its prodromal stages. Neuroimage 155, 530–548 28414186 10.1016/j.neuroimage.2017.03.057 PMC5511557

[138] Jo T (2019) Deep learning in Alzheimer’s disease: diagnostic classification and prognostic prediction using neuroimaging data. Front. Aging Neurosci 11, 220 31481890 10.3389/fnagi.2019.00220 PMC6710444

[139] Vogel JW (2021) Four distinct trajectories of tau deposition identified in Alzheimer’s disease. Nat. Med 27, 871–881 33927414 10.1038/s41591-021-01309-6 PMC8686688

[140] Toschi N (2019) Biomarker-guided clustering of Alzheimer’s disease clinical syndromes. Neurobiol. Aging 83, 42–53 31585366 10.1016/j.neurobiolaging.2019.08.032

[141] Riedel BC (2018) Uncovering biologically coherent peripheral signatures of health and risk for Alzheimer’s disease in the aging brain. Front. Aging Neurosci 10, 390 30555318 10.3389/fnagi.2018.00390 PMC6283260

[142] Chekroud AM (2016) Cross-trial prediction of treatment outcome in depression: a machine learning approach. Lancet Psychiatry 3, 243–250 26803397 10.1016/S2215-0366(15)00471-X

[143] Hampel H (2020) A precision medicine framework using artificial intelligence for the identification and confirmation of genomic biomarkers of response to an Alzheimer’s disease therapy: analysis of the blarcamesine (ANAVEX2–73) Phase 2a clinical study. Alzheimers Dement. (N Y) 6, e12013 32318621 10.1002/trc2.12013 PMC7167374

[144] Wilkinson J (2020) Time to reality check the promises of machine learning-powered precision medicine. Lancet Digit. Health 2, e677–e680 33328030 10.1016/S2589-7500(20)30200-4 PMC9060421

[145] Boustani M (2020) Passive digital signature for early identification of Alzheimer’s disease and related dementia. J. Am. Geriatr. Soc 68, 511–518 31784987 10.1111/jgs.16218

[146] Ben Miled Z (2020) Predicting dementia with routine care EMR data. Artif. Intell. Med 102, 101771 31980108 10.1016/j.artmed.2019.101771

[147] Jimenez-Luna J (2020) Drug discovery with explainable artificial intelligence. Nat. Mach. Intell 2, 573–584

[148] Jobin A (2019) The global landscape of AI ethics guidelines. Nat. Mach. Intell 1, 389–399

[149] Rudin C (2019) Stop explaining black box machine learning models for high stakes decisions and use interpretable models instead. Nat. Mach. Intell 1, 206–215 35603010 10.1038/s42256-019-0048-x PMC9122117

[150] Dinsdale NK (2022) Challenges for machine learning in clinical translation of big data imaging studies. Neuron 110, 3866–3881 36220099 10.1016/j.neuron.2022.09.012

[151] Menardi A (2022) Toward noninvasive brain stimulation 2.0 in Alzheimer’s disease. Ageing Res. Rev 75, 101555 34973457 10.1016/j.arr.2021.101555 PMC8858588

[152] Jack CR Jr. (2018) NIA-AA research framework: toward a biological definition of Alzheimer’s disease. Alzheimers Dement. 14, 535–562 29653606 10.1016/j.jalz.2018.02.018 PMC5958625

[153] Ferretti MT (2018) Sex differences in Alzheimer disease - the gateway to precision medicine. Nat. Rev. Neurol 14, 457–469 29985474 10.1038/s41582-018-0032-9

[154] Jack CR Jr. (2016) A/T/N: An unbiased descriptive classification scheme for Alzheimer disease biomarkers. Neurology 87, 539–547 27371494 10.1212/WNL.0000000000002923 PMC4970664

[155] Seyfried NT (2017) A multi-network approach identifies protein-specific co-expression in asymptomatic and symptomatic Alzheimer’s disease. Cell Syst. 4, 60–72 27989508 10.1016/j.cels.2016.11.006 PMC5269514

[156] Leng F and Edison P (2021) Neuroinflammation and microglial activation in Alzheimer disease: where do we go from here? Nat. Rev. Neurol 17, 157–172 33318676 10.1038/s41582-020-00435-y

[157] Hampel H (2020) A path toward precision medicine for neuroinflammatory mechanisms in Alzheimer’s disease. Front. Immunol 11, 456 32296418 10.3389/fimmu.2020.00456 PMC7137904

[158] Zhao P (2022) A tetravalent TREM2 agonistic antibody reduced amyloid pathology in a mouse model of Alzheimer’s disease. Sci. Transl. Med 14, eabq0095 36070367 10.1126/scitranslmed.abq0095

[159] Beam E (2021) A data-driven framework for mapping domains of human neurobiology. Nat. Neurosci 24, 1733–1744 34764476 10.1038/s41593-021-00948-9 PMC8761068

[160] Sabbagh MN (2020) Early detection of mild cognitive impairment (MCI) in an at-home setting. J. Prev. Alzheimers Dis 7, 171–178 32463070 10.14283/jpad.2020.22

[161] Konig A (2015) Automatic speech analysis for the assessment of patients with predementia and Alzheimer’s disease. Alzheimers Dement. (Amst) 1, 112–124 27239498 10.1016/j.dadm.2014.11.012 PMC4876915

[162] Oyama A (2019) Novel method for rapid assessment of cognitive impairment using high-performance eye-tracking technology. Sci. Rep 9, 12932 31506486 10.1038/s41598-019-49275-x PMC6736938

[163] David R (2012) Decreased daytime motor activity associated with apathy in Alzheimer disease: an actigraphic study. Am. J. Geriatr. Psychiatry 20, 806–814 21997602 10.1097/JGP.0b013e31823038af

[164] Hampel H (2023) Biological mechanism-based neurology and psychiatry: a BACE1/2 and downstream pathway model. Curr. Neuropharmacol 21, 31–53. 10.2174/1570159×19666211201095701 34852743 PMC10193755

[165] Zhao S and Iyengar R (2012) Systems pharmacology: network analysis to identify multiscale mechanisms of drug action. Annu. Rev. Pharmacol. Toxicol 52, 505–521 22235860 10.1146/annurev-pharmtox-010611-134520 PMC3619403

[166] Geerts H (2020) Quantitative systems pharmacology for neuroscience drug discovery and development: current status, opportunities, and challenges. CPT Pharmacometrics Syst. Pharmacol 9, 5–20 31674729 10.1002/psp4.12478 PMC6966183

[167] Ferretti MT (2019) Account for sex in brain research for precision medicine. Nature 569, 40 10.1038/d41586-019-01366-5 31040416

[168] Meoni S (2020) Sex differences in movement disorders. Nat. Rev. Neurol 16, 84–96 31900464 10.1038/s41582-019-0294-x

[169] Jacobs GR (2019) Developmentally divergent sexual dimorphism in the cortico-striatal-thalamic-cortical psychosis risk pathway. Neuropsychopharmacology 44, 1649–1658 31060043 10.1038/s41386-019-0408-6 PMC6785143

[170] Vergallo A (2019) Brain Abeta load association and sexual dimorphism of plasma BACE1 concentrations in cognitively normal individuals at risk for AD. Alzheimers Dement. 15, 1274–1285 31627825 10.1016/j.jalz.2019.07.001

[171] Hohman TJ (2018) Sex-specific association of apolipoprotein E with cerebrospinal fluid levels of tau. JAMA Neurol. 75, 989–998 29801024 10.1001/jamaneurol.2018.0821 PMC6142927

[172] Cavedo E (2018) Sex differences in functional and molecular neuroimaging biomarkers of Alzheimer’s disease in cognitively normal older adults with subjective memory complaints. Alzheimers Dement. 14, 1204–1215 30201102 10.1016/j.jalz.2018.05.014

[173] Babapour Mofrad R and van der Flier WM (2019) Nature and implications of sex differences in AD pathology. Nat. Rev. Neurol 15, 6–8 30542072 10.1038/s41582-018-0115-7

[174] Christensen A and Pike CJ (2015) Menopause, obesity and inflammation: interactive risk factors for Alzheimer’s disease. Front. Aging Neurosci 7, 130 26217222 10.3389/fnagi.2015.00130 PMC4493396

[175] Hampel H (2018) Precision medicine and drug development in Alzheimer’s disease: the importance of sexual dimorphism and patient stratification. Front. Neuroendocrinol 50, 31–51 29902481 10.1016/j.yfrne.2018.06.001

[176] Frohlich H (2018) From hype to reality: data science enabling personalized medicine. BMC Med. 16, 150 30145981 10.1186/s12916-018-1122-7 PMC6109989

[177] Gibbs RM (2018) Toward precision medicine for neurological and neuropsychiatric disorders. Cell Stem Cell 23, 21–24 29887317 10.1016/j.stem.2018.05.019

[178] Denny JC and Collins FS (2021) Precision medicine in 2030-seven ways to transform healthcare. Cell 184, 1415–1419 33740447 10.1016/j.cell.2021.01.015 PMC9616629

[179] Beger RD (2016) Metabolomics enables precision medicine: “a white paper, community perspective”. Metabolomics 12, 149 27642271 10.1007/s11306-016-1094-6 PMC5009152

[180] Consortium GTEx (2013) The Genotype-Tissue Expression (GTEx) project. Nat. Genet 45, 580–585 23715323 10.1038/ng.2653 PMC4010069

[181] Althoff T (2017) Large-scale physical activity data reveal worldwide activity inequality. Nature 547, 336–339 28693034 10.1038/nature23018 PMC5774986

[182] All of Us Research Program Investigators (2019) The “All of Us” research program. N. Engl. J. Med 381, 668–676 31412182 10.1056/NEJMsr1809937 PMC8291101

[183] Sudlow C (2015) UK biobank: an open access resource for identifying the causes of a wide range of complex diseases of middle and old age. PLoS Med. 12, e1001779 25826379 10.1371/journal.pmed.1001779 PMC4380465

[184] Smith RA (2019) Cancer screening in the United States, 2019: a review of current American Cancer Society guidelines and current issues in cancer screening. CA Cancer J. Clin 69, 184–210 30875085 10.3322/caac.21557

[185] Heitzer E (2019) Current and future perspectives of liquid biopsies in genomics-driven oncology. Nat. Rev. Genet 20, 71–88 30410101 10.1038/s41576-018-0071-5

[186] Kumar-Sinha C and Chinnaiyan AM (2018) Precision oncology in the age of integrative genomics. Nat. Biotechnol 36, 46–60 29319699 10.1038/nbt.4017 PMC6364676

[187] Fitzgerald RC (2020) Big data is crucial to the early detection of cancer. Nat. Med 26, 19–20 31932790 10.1038/s41591-019-0725-7

[188] Drilon A (2018) Efficacy of larotrectinib in TRK fusion-positive cancers in adults and children. N. Engl. J. Med 378, 731–739 29466156 10.1056/NEJMoa1714448 PMC5857389

